# Marine alkaloids as bioactive agents against protozoal neglected tropical diseases and malaria†

**DOI:** 10.1039/d0np00078g

**Published:** 2021-04-12

**Authors:** Andre G. Tempone, Pauline Pieper, Samanta E. T. Borborema, Fernanda Thevenard, Joao Henrique G. Lago, Simon L. Croft, Edward A. Anderson

**Affiliations:** Centre for Parasitology and Mycology, Instituto Adolfo Lutz São Paulo 01246-000 Brazil andre.tempone@ial.sp.gov.br; Chemistry Research Laboratory, University of Oxford 12 Mansfield Road Oxford OX1 3TA UK edward.anderson@chem.ox.ac.uk; Centre of Natural Sciences and Humanities, Federal University of ABC Sao Paulo 09210-580 Brazil; Faculty of Infectious and Tropical Diseases, London School of Hygiene and Tropical Medicine London WC1E 7HT UK simon.croft@lshtm.ac.uk

## Abstract

Covering: 2000 up to 2021

Natural products are an important resource in drug discovery, directly or indirectly delivering numerous small molecules for potential development as human medicines. Among the many classes of natural products, alkaloids have a rich history of therapeutic applications. The extensive chemodiversity of alkaloids found in the marine environment has attracted considerable attention for such uses, while the scarcity of these natural materials has stimulated efforts towards their total synthesis. This review focuses on the biological activity of marine alkaloids (covering 2000 to up to 2021) towards Neglected Tropical Diseases (NTDs) caused by protozoan parasites, and malaria. Chemotherapy represents the only form of treatment for Chagas disease, human African trypanosomiasis, leishmaniasis and malaria, but there is currently a restricted arsenal of drugs, which often elicit severe adverse effects, show variable efficacy or resistance, or are costly. Natural product scaffolds have re-emerged as a focus of academic drug discovery programmes, offering a different resource to discover new chemical entities with new modes of action. In this review, the potential of a range of marine alkaloids is analyzed, accompanied by coverage of synthetic efforts that enable further studies of key antiprotozoal natural product scaffolds.

## Abstract 2

This Review discusses the isolation and bioactivity of marine alkaloids against protozoan parasite diseases, and chemical syntheses that enable the further development of these scaffolds as drug leads.

## Graphical abstract

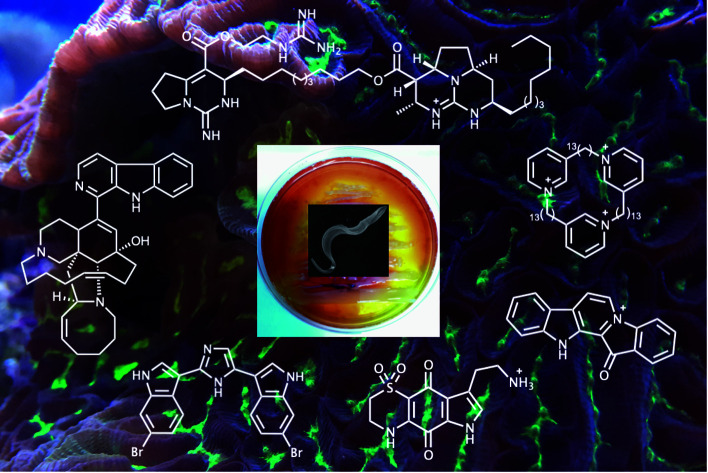

## 1. Introduction

Malaria, human African trypanosomiasis (sleeping sickness), Chagas disease and leishmaniasis are infectious diseases caused by protozoan parasites, which are transmitted to humans by insect vectors. Two of these diseases (malaria and leishmaniasis) occur worldwide,^1,2^ while the distributions of Chagas disease and human African trypanosomiasis (HAT) are mainly confined to Central/South America^3^ and sub-Saharan Africa, respectively.^4^ Over the past decade, another terminology for these diseases has become widely used: malaria is referred to as a ‘disease of poverty’, while leishmaniasis, Chagas disease and HAT are described as Neglected Tropical Diseases (NTDs),^5^ which refers to diseases of poverty where both the patients and the diseases themselves have typically received less funding, and suffered from a lower public health profile. For NTDs in particular, there is an urgent need for new therapies to combat the drawbacks associated with existing treatments, which include adverse side effects (often leading to patient compliance issues), limited efficacy or emerging resistance and, in certain cases, high cost and prolonged treatment regimens.^6–8^ These challenges are compounded by socio-economic consequences for affected nations, which are often among the least developed.^9^ While drug repurposing can offer one avenue to new treatments,^10–12^ natural products are also re-emerging as candidates for the discovery of new anti-protozoal agents,^13^ and indeed have a proven track record as therapeutic agents against protozoal diseases.^14,15^ Traditionally regarded as non-viable for such purposes due to synthetic intractability, low natural abundance, polypharmacology, and inflexibility with respect to analogue synthesis, advances in synthetic organic chemistry offer a realistic means to access and diversify such scaffolds.^16,17^ This review focuses on alkaloids isolated from the marine environment, which have long been recognised as a low-abundance but high-potential source of bioactive agents. Following a discussion of the existing state of the art with respect to the diseases and their treatments, those marine alkaloids that have been evaluated for anti-protozoal bioactivity in the period 2000–2020 are described, with an emphasis on the most potent isolates. We then discuss synthetic approaches to marine alkaloids, emphasising routes that offer the most efficient access to the important bioactive alkaloid scaffolds, and those that are well-suited to analogue design and synthesis.

## 2. Protozoal parasite diseases

Among protozoal parasitic diseases, malaria has received the most attention due to its widespread nature and impact on human society. It is caused by five species of parasites from the genus *Plasmodium*, with 219 million cases and ∼500 000 deaths in 2017 alone (most in sub-Saharan Africa); the species that results in most fatality is *P. falciparum*. The main cause of acute malaria is the erythrocytic stage of the parasite which multiplies rapidly, resulting in up to 200 million cases per year and causing over 400 000 deaths.^18^ This stage has consequently been the main target for clinical intervention and hence the focus of most drug discovery and development efforts.

Following the bite of an infected anopheline mosquito, the sporozoite form of the *Plasmodium* parasite invades liver cells (hepatocytes) where it divides, releasing merozoite forms that infect erythrocytes. The liver stage in *P. falciparum* infections is the target for prophylactic drugs, and is an important focus for *P. vivax* cure where there is persistent liver infection, including a dormant hypnozoite form. Maturation of the merozoites into the sexual gametocyte stage of the parasite in the erythrocytes offers a further drug target; this is the form that is infective to the *Anopheles* mosquito vector where fusion of gametes eventually generates further infective sporozoites. Drugs targeting this stage are ‘altruistic’ drugs – they will not cure the patient but will prevent transmission. Due to the potential to target three different stages of the parasite life cycle, research is now focused on developing combination therapies of drugs with matched for pharmacokinetic profiles;^19,20^ indeed, the use of combination therapies for malaria treatment has been a WHO policy requirement since 2001 as a result of the rapid evolution of resistant *Plasmodium* forms using monotherapies. Drug resistance,^21^ observed for chloroquine and pyrimethamine as early as the 1960s, has since been a driver of antimalarial drug research and development, with major initiatives in the USA (resulting in mefloquine) and China (resulting in artemisinin). The establishment of the Medicines for Malaria Venture (MMV) public–private partnership has stimulated the development of a large portfolio of novel compounds, with over 20 active projects at stages from discovery to clinical trials.^22^

Human African trypanosomiasis (HAT), a disease transmitted by tsetse flies, is caused by trypanosomatid protozoan parasites that have flagella and other unique organelles. Two species cause this disease: *Trypanosoma brucei gambiense* (anthroponotic and distributed in West and Central Africa) and *T. b. rhodesiense* (zoonotic and found mainly in Central and East Africa). The initial acute infection is haemo-lymphatic (stage 1), developing after several months into chronic “sleeping sickness” through invasion of the central nervous system (CNS, stage 2). Over the past decade the number of cases of HAT has dropped significantly from over 10 000 to just under 1000 thousand cases per annum,^23^ and elimination of HAT is now a realistic target. A major challenge for drug discovery for HAT is the design of compounds that can cross the blood–brain barrier to achieve a sufficiently high concentration in the CNS to kill the parasites. Until recently, treatment of CNS infections involved the use of arsenic-based drugs (*e.g.* melarsoprol). However, in the past decade the Drugs for Neglected Diseases initiative (DNDi) has developed fexinidazole, the first oral treatment for HAT.^24^ Another potential therapy, acoziborole, is in a clinical trial as a potential single dose treatment,^24^ while other lead candidates have been identified that may offer new opportunities.^25^

Chagas disease, which is caused by another trypanosomatid protozoan – *Trypanosoma cruzi* – is transmitted by triatomine bugs and is both anthroponotic and zoonotic. Upon infection, the parasite invades many different tissues, dividing as the intracellular amastigote form in the cell cytoplasm. As the disease progresses towards the chronic stage, the parasites appear to go into a latent phase, and for many patients there can be a 10–30 year period before the potentially fatal chronic disease appears (with cardiac, neurological and intestinal complications). An estimated 6–7 million people are infected with this parasite, with over 10 000 deaths per annum. Drug development faces the challenges of identifying compounds that kill both dividing amastigotes and non-dividing trypomastigotes coming from a number of taxonomic units, with pharmacokinetic properties that ensure distribution to many tissue types. There are currently two drugs available to treat Chagas disease: the nitrofuran nifurtimox, and the nitroimidazole benznidazole.^26,27^ Both are oral drugs, but require long courses of treatment and can have profound side effects and show variable efficacy. Azoles, as sterol biosynthesis inhibitors, were also pursued as potential treatments for several decades, but have shown limited efficacy in clinical trials. Recently the DNDi has identified the potential of fexinidazole, which has recently been evaluated in a Phase II proof-of-concept study, and has also shown how shorter courses of benznidazole can be better used to treat chronic infections.^28^

Leishmaniasis is a disease complex, with up to 20 different species of *Leishmania* that are transmitted by female phlebotomine sandflies. There are several clinically distinct manifestations, which are broadly classified as either the potentially fatal visceral leishmaniasis (VL), or the self-curing but disfiguring cutaneous leishmaniasis (CL). There are over 100 000 cases of the mainly anthroponotic VL per annum, with the main foci being in East Africa and the Indian sub-continent (ISC). In the ISC VL is the subject of an elimination programme, and there are now less than 5000 cases per annum. In contrast, some 1–2 million cases per annum of the mainly zoonotic cutaneous disease (CL) are reported. In mammalian hosts, the parasites survive and multiply as the amastigote form within macrophages. The challenge for drug development has been to identify compounds active against all or most of the ∼20 species that cause disease in humans, with pharmacokinetic properties that enable distribution to either the liver, spleen, and bone marrow for VL, or the skin for CL. Complex skin manifestations (such as mucocutaneous and diffuse cutaneous leishmaniasis) as well as VL/HIV co-infections add to the challenges for new treatments. Two therapeutic agents introduced during the past 15 years have made an impact on visceral leishmaniasis: miltefosine (an alkylphospholipid administered orally), and a unilamellar liposomal formulation of the polyketide natural product amphotericin B (AmBisome®). These two drugs have largely replaced the traditional pentavalent antimonials for VL,^29^ but not for CL where treatment is still dependent on these toxic heavy metal complexes. Topical formulations have also been a focus for CL, including several of the aminoglycoside paromomycin. New candidates for VL treatment are in development:^26,27^ six clinical and pre-clinical candidates have been disclosed by the DNDi, with collaboration from two pharmaceutical companies (GSK and Novartis).^30,31^ Some of these compounds also show potential for CL.^32^

## 3. Antiprotozoal marine alkaloids

Alkaloids comprise an extensive and important group of secondary metabolites produced by plants, microorganisms, marine organisms, insects, and animals.^33,34^ As many alkaloids likely evolved as chemical defences for marine organisms, the origin of their bioactivity against human pathogens is a complex question. Possible factors include conserved morphology of binding surfaces between proteins of different sequence and function, genetic homology between different organisms, and even the biosynthesis of natural products itself, which requires intermediates to be successfully processed by a number of enzymes.^35^ One further complication, and potential benefit, is that some natural products have been shown to be biosynthesized by a co-habiting microorganism. Nonetheless, the structural diversity of these compounds has provided access to new areas of chemical space, and the consequent discovery of activity against human diseases.^36,37^

**Fig. 1:**
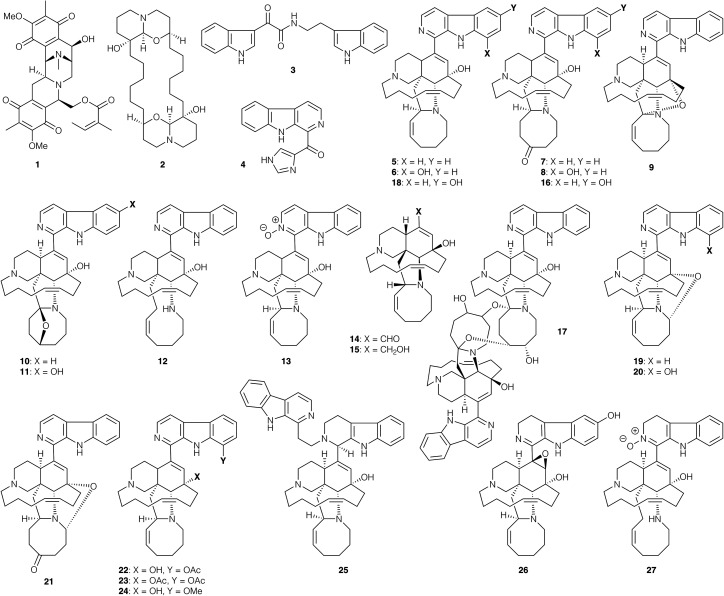
Natural products and semi-synthetic derivatives 1–27.

**Fig. 2:**
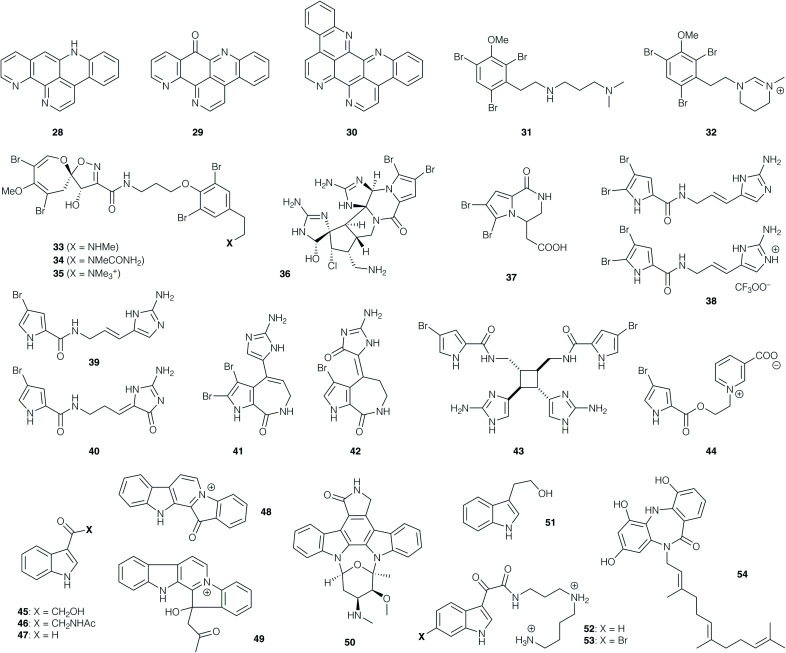
Natural products and synthetic derivatives 28–54.

**Fig. 3:**
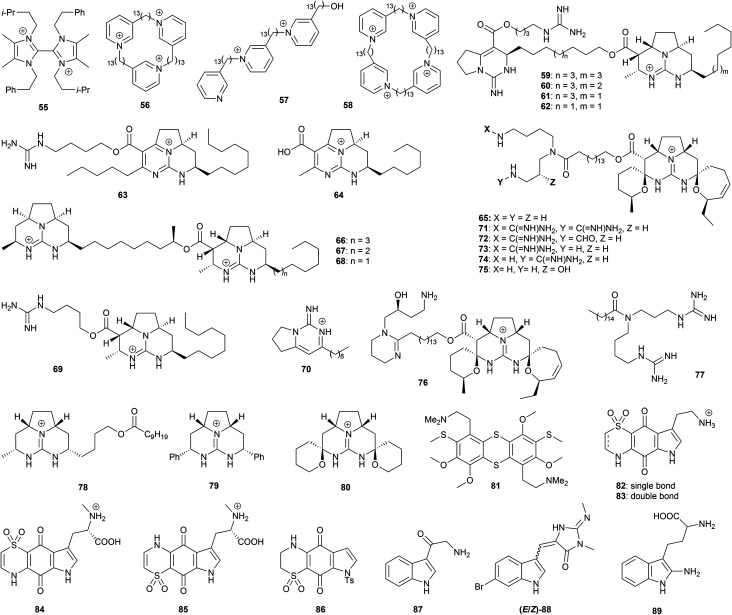
Natural products and synthetic derivatives 55–88.

**Fig. 4:**
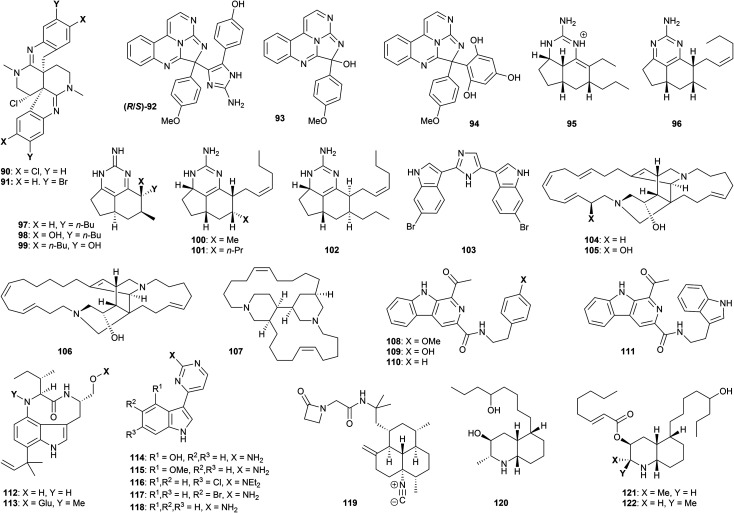
Natural products and synthetic derivatives 93–122.

While the biological potential of certain alkaloids against protozoan diseases has been covered previously,^38,39^ this review focuses specifically on alkaloids isolated from marine organisms and microorganisms (Fig. 1–4). In discussing these natural products, selected semi-synthetic and synthetic derivatives are also included for relevance of bioactivity (see the ESI† for tabulation of natural products, marine source, and bioactivity data, including a ranking of the most active and selective compounds). For the information of the reader, the terminology of compound activities is defined in Box 1, and the nature of the phenotypic assays used for each parasite (and its different forms where relevant) is outlined in Box 2. Box 3 summarises some of the most critical accepted criteria for ‘hit’ and ‘lead’ compound identification against *P. falciparum*, *T. cruzi* and *L. infantum*/*L. donovani*, as defined by the Japanese Global Health Innovative Technology, in collaboration with the MMV and the DNDi.^40^

The survey of natural products begins with the alkaloid renieramycin A (1, Fig. 1), which was isolated *via* bioactivity-guided fractionation of the lipophilic extract from the marine sponge *Neopetrosia* sp. Renieramycin A displayed an IC_50_ value of 0.2 μg mL^−1^ against *L. amazonensis* promastigotes and a 50% cytotoxicity concentration (CC_50_) against mammalian cells of 2.2 μg mL^−1^.^43^ Araguspongin C (2), isolated from the MeOH extract of the sponge *Haliclona exigua*,^44^ is the sole member of a large family of macrocyclic bis-*N*,*O*-acetal containing alkaloids that has been screened for antiprotozoal activity; it shows *in vitro* activity against *L. donovani* intracellular amastigotes (48% inhibition at 100 μg mL^−1^) and no mammalian cytotoxicity up to 100 μg mL^−1^. When administered in *L. donovani*-infected hamsters, 2 reduced the parasite burden by 39%.^45^

Box 1: Terminology of drug activities in experimental models.

IC_50_ and IC_90_ refer to concentrations that inhibit growth of parasites cultures by 50% and 90%, normally calculated from a dose–response curve. Some authors use the terms EC_50_ and EC_90_, effective concentrations, expressing similar activities.

CC_50_ and CC_90_ refer to concentrations that indicate cytotoxic activity against mammalian cell lines. In this context the selectivity index (SI) is the ratio of between activity against the mammalian cells (CC_50_) and the protozoan parasites (EC_50_).

ED_50_ and ED_90_ refer to the effective doses that reduce parasite load by 50% and 90% in *in vivo* studies, normally calculated from dose–response curves. Reduction in parasitaemia refers only to the blood infection.

Box 2: *In vitro* phenotypic assays to determine compound activities.

Data referred to in this review is derived mainly from *in vitro* assays, which have been described in detail elsewhere for *Plasmodium*^41^ and *Trypanosoma* and *Leishmania*.^42^

*Plasmodium*: most assays are based on intra-erythrocytic cultures of *Plasmodium falciparum* strains with compound exposure over 48 hours.

*Trypanosoma brucei*: assays are based on cultures of extracellular trypomastigotes of *T. brucei* spp. with compound exposure over 48 or 72 hours.

*Trypanosoma cruzi*: assays are based on either (i) culture of the extracellular epimastigote form, (ii) extracellular trypomastigotes, a non-dividing stage or (iii) the intracellular amastigote form, grown in a range of mammalian cell lines for 48 or 72 hours; this is the most relevant assay for hit identification.

*Leishmania*: assays are based on either (i) culture of the extracellular promastigote form, or (ii) the intracellular amastigote form, grown in primary isolated macrophages or macrophage cell lines for 48 or 72 hours; this is the most relevant assay for hit identification. *L. donovani*/*L. infantum* are the chosen target species for VL, and either *L. major*, *L. mexicana* and *L. braziliensis* as the target species for CL.

Box 3: General hit criteria:

The criteria listed below are as outlined in ref. 40.

Readily synthesized, not contain reactive/unstable groups in the pharmacophore, be suited for structural variation; pass drug-like filters (*e.g.* pan-assay interference, PAINS).

SI >10 between mammalian cytotoxicity (CC_50_) and EC_50_ (malaria), or IC_50_ (CD/VL).

Synthesized in ≤5 steps with an acceptable yield and solubility.

No serious IP conflicts; preliminary knowledge of the structure–activity relationship (SAR) is desirable.

General lead criteria:

High potency (IC_50_), but not at the expense of physicochemical or drug metabolism and pharmacokinetic (DMPK) properties.

Synthesis enables preparation and testing of many analogues in a short time frame.

SI >100 between mammalian cytotoxicity (CC_50_) and EC_50_ (malaria), or IC_50_ (CD/VL); no acute toxicity.

Desirable physicochemical properties (*e.g.* solubility >10 μM; log *P* <5 and ideally <3), suitable DMPK profile, oral efficacy in the relevant disease model.

Specific criteria: Malaria

Hit: EC_50_ <1 μM against sensitive and multiple resistant strains of *Plasmodium* spp.

**Early lead:** EC_50_ <100 nM for sensitive and multidrug-resistant strains of *Plasmodium* spp.

• *In vivo* efficacy criteria: depending on the stage of infection that is targeted; *e.g.* for the blood stages of infection, 90% of the target pathogen eradicated in a mouse model at <50 mg per kg, up to four doses over four days.

**Specific criteria: Chagas disease**

**Hit:** IC_50_ <10 μM against intracellular *T. cruzi*.

**Lead:** 80% reduction of parasite burden in organs or tissues in an acute mouse model, or no parasites detected at the end of treatment and an increase in lifespan, up to 10 doses at 50 mg per kg delivered orally.

**Specific criteria: Visceral leishmaniasis**

**Hit:** IC_50_ <10 μM against intracellular *Leishmania* spp.

**Lead:** >70% reduction in liver parasite burden in a mouse or hamster model, after up to 5 doses at 50 mg per kg, delivered orally once or twice per day.

The indole alkaloid 8,9-dihydrocoscinamide B (3) was isolated from the marine sponge *Conscinoderma* sp.^46^ and, being a relatively simple target, also accessed by chemical synthesis.^47^ The synthetic natural product was found to eliminate 97% of the intracellular amastigotes of *L. donovani in vitro* at 10 μg mL^−1^; other synthetic analogues exhibited activity from 59 to 97% inhibition at the same concentration.

Among marine alkaloids, the manzamine natural products are a particularly important family that has been extensively studied for their antiprotozoal properties.^48,49^ For example, the Indonesian sponge metabolites des-*N*-methylxestomanzamine A (4), manzamine A (5), 8-hydroxymanzamine A (6), manzamines E (7) and F (8), 12,34-oxamanzamine A (9), 6-deoxymanzamine X (10) and manzamine X (11) exhibited *in vitro* activity against *L. donovani* promastigotes with IC_50_ values of 0.97–35 μg mL^−1^, and CC_50_ values ranging from 4.7 μg mL^−1^ to non-cytotoxic levels (CC_50_ >4.7 μg mL^−1^).^50^ Manzamine A (5) also displayed *in vivo* activity against *P. berghei* at a single dose (murine model, 100 μmol kg^−1^), reducing the parasite load by 97% at the onset of the parasitaemia.^51^5 also prolonged the survival of treated animals by promoting the production of specific antibodies (IgG) against the *Plasmodium* parasite. Manzamine alkaloids were also isolated from the sponge *Acanthostronglyophora* sp., namely manzamine J (12), manzamine A *N*-oxide (13), ircinal A (14), ircinol A (15), 6-hydroxymanzamine E (16) and neo-kauluamine (17). These presented leishmanicidal activity against *L. donovani* promastigotes with IC_50_ values of 0.9–25.0 μg mL^−1^, and CC_50_ values ranging from 1.1 μg mL^−1^ to non-cytotoxic concentrations (CC_50_ >4.7 μg mL^−1^).^52^ Further studies with the same sponge afforded manzamine Y (18), 12,28-oxamanzamine A (19), 12,28-oxa-8-hydroxymanzamine A (20) and 12,28-oxamanzamine E (21), which led to comparable IC_50_ values of 1.6–24.0 μg mL^−1^, and CC_50_ values ranging from 1.1 μg mL^−1^ to non-cytotoxic concentrations (CC_50_ >4.7 μg mL^−1^).^53^ Semi-synthetic derivatives 8-acetoxymanzamine A (22), 8,12-diacetoxymanzamine A (23) and 8-methoxymanzamine A (24) exhibited IC_50_ values against *L. donovani* promastigotes of 3.4–16.0 μg mL^−1^, and 30–1200 ng mL^−1^ against chloroquine-resistant *P. falciparum* (W2 clone).^54^ Notably, the relatively low mammalian cytotoxicity of these compounds (CC_50_ values of 370 μg mL^−1^ to non-cytotoxic) afforded a high selectivity index. *In vivo* studies with 22 in *Plasmodium*-infected mice led to a reduction of parasitaemia of 84% after 10 days and 48% after 14 days at 30 mg kg^−1^ (3 oral doses). Finally, a study of the sponge *Amphimedon* sp. afforded the manzamine relative zamamidine C (25), 3,4-dihydro-6-hydroxy-10,11-epoxymanzamine A (26) and 3,4-dihydromanzamine J *N*-oxide (27), which displayed IC_50_ values of 0.04–4.40 μg mL^−1^ for *T. b. brucei*, and 0.58–7.00 μg mL^−1^ for *P. falciparum*.^49^ Although no mammalian cytotoxicity studies were performed, an anticancer assay with KB human epidermoid carcinoma cells demonstrated IC_50_ values of 17.7, 7.2, and 6.7 μg mL^−1^ respectively. From these various studies of the manzamine family, the most potent activities against *L. donovani* (∼1 μg mL^−1^) were obtained for manazmine A (5), manazmine A *N*-oxide (13), ircinol A (15) and manzamine Y (18), several of which have subsequently been accessed by chemical synthesis (*vide infra*). It is interesting that ircinol A is both *pseudo*-enantiomeric to the majority of the manzamine family, and lacks the typical carboline sidechain, and yet maintains high levels of antiprotozoal activity; this may enable simplification of in the design of manzamine analogues.

In contrast to the structural complexity of the manzamines, 12-deoxyascididemin (28, Fig. 2), ascididemin (29) and eilatin (30) comprise a small group of relatively simple polycyclic phenanthrolines isolated from the ascidian *Polysyncraton echinatum*. These alkaloids exhibited potent antitrypanosomal activity against *T. b. brucei* with IC_50_ values of 0.07, 0.03 and 1.30 μM respectively, and CC_50_ values of 76, 1.4 and 62 μM (corresponding to a promising selectivity index range of 46–99).^55^ While the parent polyaromatic structures may exhibit undesirable physicochemical properties, their structural simplicity renders them attractive targets for synthesis.

Certain families of marine alkaloids exhibit extensive bromination, such as the dibromoarene-containing convolutamines I (31) and J (32), which were isolated from the hexane extract of the bryozoan *Amathia tortusa*.^56^ These compounds were evaluated against *T. b. brucei*, with 31 showing the higher potency (with an IC_50_ value of 1.1 μM), but also higher toxicity against human embryonic kidney cells (32 was non-toxic). The related bromospiroacetal psammaplysin F (33), isolated from *Hyatella* sp., showed an IC_50_ value of 5.6 μM against *T. cruzi*,^57^ but a moderate selectivity index. A further isolation from the dichloromethane/methanol extract of the sponge *Pseudoceratina* sp. afforded 33 along with psammaplysins G (34)^58^ and H (35).^59^ Compound 33 displayed activity against chloroquine-sensitive and resistant strains of *P. falciparum* (IC_50_ values of 1.4 and 0.87 μM, respectively), with a mammalian cytotoxicity value of CC_50_ 11 μM.^58^ Psammaplysin H (35) showed the most potent IC_50_ value of 0.4 μM against the *P. falciparum* strain 3D7, while 34 displayed an IC_50_ value of 5.2 μM. Importantly, 35 also displayed no toxicity to mammalian cells to the highest tested concentration of 40 μM, while 33 and 34 showed mammalian cytotoxicity below 20 μM.^59^

Bromopyrrole natural products represent an extensive family of marine alkaloids that have attracted attention both from a bioactivity and synthetic perspective. For instance, dibromopalauamine (36), longamide B (37), oroidin (38), hymenidin (39), dispacamide B (40), stevensine (41), spongiacidin B (42), sceptrin (43), and agelongine (44) were isolated from marine sponges of the *Agelas* and *Axinella* genera;^60^ evaluation of their *in vitro* activity against *T. b. rhodesiense* and *L. donovani* revealed IC_50_ values of 0.4 and 1.1 μg mL^−1^ respectively for 36, while 37 displayed IC_50_ values of 1.5 and 3.8 μg mL^−1^ respectively, although both compounds possessed some toxicity against mammalian cells. The other active compounds in this collection displayed antiprotozoal activities against *T. b. rhodesiense* with IC_50_ values between 9.7 and 78 μM, and against *L. donovani* with IC_50_ values between 16 and 77 μM. Compounds 42, 40 and 36 proved the most active compounds against *P. falciparum* (with IC_50_ values of 1.1, 1.3 and 1.5 μM respectively); the other active compounds afforded values between 3.9 and 12.5 μM.^60^ The anti-plasmodial activity of oroidin (38) potentially arises from inhibition of the *P. falciparum* enoyl-ACP reductase (PfFabI) enzyme (IC_50_ 0.30 μg mL^−1^ = 0.77 μM).^61^

Numerous indole alkaloids have been isolated from marine sources. Bioactivity-guided fractionation of the EtOAc extract of the marine bacterium *Bacillus pumilus*, collected from the black coral *Antipathes* sp., led to isolation of 3-hydroxyacetylindole (45), acetyl-β-oxotryptamine (46), and 3-formylindole (47). These compounds inhibited *T. cruzi* amastigotes with IC_50_ values of 19.4–26.9 μM, but showed no activity against *P. falciparum* or *L. donovani*. 46 and 47 showed cytotoxicity for African green monkey kidney cells (CC_50_ values of 66 and 87 μM, respectively).^62^

The polycyclic indoles fascaplysin (48) and homofascaplysin (49) were obtained from the dichloromethane/MeOH extract of the sponge *Hyrtios cf. erecta*, and were found to exhibit impressive levels of antiprotozoal activity.^63^48 showed *in vitro* activity against *T. b. rhodesiense* (IC_50_ 0.17 μg mL^−1^) and against the K1 and NF54 strains of *P. falciparum* (IC_50_ 50 and 34 ng mL^−1^ respectively), while 49 exhibited IC_50_ values of 14 and 24 ng mL^−1^ respectively against the latter. With CC_50_ values of 1.1 and 2.5 μg mL^−1^ respectively, both compounds displayed promising selectivity indices; together with their structural accessibility, this has rendered them popular targets for total synthesis (*vide infra*). This is similarly the case for the indolocarbazole alkaloid staurosporine (50), isolated from the sponge associated actinomycetes *Streptomyces* sp.; this compound showed activity against *L. major* promastigotes (IC_50_ of 5.3 μM), and *T. b. brucei* (IC_50_ of 20 nM, with a CC_50_ value of 1.3 μM against 293T kidney epithelial cells; SI of 65).^64^

The rather simpler indole alkaloid tryptophol (51), obtained from sponge species *Spongia* sp. and *Ircinia* sp., exhibited activity against *L. donovani* amastigotes (IC_50_ 9.6 μg mL^−1^), *T. cruzi* amastigotes (IC_50_ 49 μg mL^−1^) and *T. b. rhodesiense* trypomastigotes (IC_50_ of 5.9 μg mL^−1^), with CC_50_ values of 63 μg mL^−1^.^65^ The indole spermidine alkaloids didemnidines A (52) and B (53), isolated from the ascidian *Didemnum* sp., were also prepared by chemical synthesis; these compounds (and synthetic intermediates *en route*) exhibited no activity against *L. donovani* amastigotes, but were moderately active against *T. cruzi* amastigotes (IC_50_ 28–130 μM) and *T. b. rhodesiense* trypomastigotes (IC_50_ 9.9–59.0 μM).^66^ Evaluation against the K1 strain of *P. falciparum* afforded IC_50_ values between 8.4 and 41 μM, but mammalian cytotoxicity was significant, with CC_50_ values around 25 μM.

Moving away from indole derivatives, diazepinomicin (54) is an interesting dibenzodiazepine obtained from the *Micromonospora* sp. RV115 strain isolated from the sponge *Aplysina aerophoba*.^67^ When tested against *T. b. brucei* trypomastigotes, 54 displayed an IC_50_ value of 13.6 μM, with no toxicity to human kidney cells HK-2 at 25 μM. The natural product was found to inhibit the *T. b. brucei* protease rhodesain.

Quaternized aromatic azacycles feature prominently in marine alkaloids, and often exhibit useful antiprotozoal activity. A study with *Paenibacillus* sp. strain DE2SH, isolated from mangrove rhizosphere soils in Ghanaian wetlands, afforded the bis-imidazolium alkaloid paenidigyamycin A (55, Fig. 3).^68^ This compound displayed IC_50_ values of 0.75 μM (*L. major* promastigotes), 7.0 μM (*L. donovani* promastigotes), 0.78 μM (*T. b. brucei* trypomastigotes), and 9.1 μM (*P. falciparum*). Natural and synthetic pyridinium ions have also been studied for antiprotozoal properties. The arctic sponge *Haliclona viscosa* has afforded a number of alkylpyridinium alkaloids such as viscosamine (56);^69^ in the course of synthetic studies towards 56, a number of 2-tridecylpyridinium alkaloids were prepared and evaluated for antiprotozoal activity alongside 56, including natural product mimics such as 57 and 58.^70^ These compounds showed antitrypanosomal potential against *T. brucei* (IC_50_ of the synthetic analogues 0.014 to 16 μM), and antileishmanial potential against *L. major* promastigotes (IC_50_ 0.032–50 μM) and *L. mexicana* amastigotes (IC_50_ 0.19–50 μM). Potent antimalarial activity was also observed, with IC_50_ values of 0.053–7.5 μM against *P. falciparum*. The mammalian cytotoxicity against HEK293 cells resulted in CC_50_ values of 11–204 μM, suggesting promising selectivity indexes, especially for *T. brucei*.

An extensive range of pyrimidine and guanidine alkaloids have been isolated from marine sources. For example, the sponge *Monanchora arbuscula* yielded batzelladine A (59), norbatzelladine A (60), dinorbatzelladine A (61), dinordehydrobatzelladine B (62), dihomodehydrobatzelladine C (63), clathriadic acid (64), ptilomycalin A (65) batzelladine L (66) and norbatzelladine L (67), which were tested for antiplasmodial activity, with many exhibiting high potency.^71^ Ptilomycalin A (65) was found to be the most active against *P. falciparum*, with an IC_50_ value of 0.1 μM, while norbatzelladines A and L (60 and 67) and batzelladines A and L (59 and 66) showed activities in the range of 0.2–0.4 μM; 62–64 displayed IC_50_ values of 0.8–4.5 μM. Batzelladine L (66), norbatzelladine L (67), batzelladine F (68), batzelladine D (69) and monalidine A (70) afforded IC_50_ values of 2–64 μM for *T. cruzi* trypomastigotes, and 2–4 μM for *L. infantum* promastigotes;^72^ the lack of a second tricyclic guanidinium ion in batzelladine D (69) appears highly detrimental to *T. cruzi* activity, but makes little difference for *L. infantum*. In terms of mechanism of action, batzelladine L and norbatzelladine L (66 and 67) caused an increase in the permeability of the *Leishmania* plasma membrane, and depolarization of the mitochondrial membrane. 66 in particular induced a significant increase in the production of reactive oxygen species (ROS), a potential pathway to cell death.^72^ The mammalian cytotoxicity against monkey kidney cells LLC-MK2 ranged from 22–130 μM. The dichloromethane/MeOH extract of the marine sponge *Monanchora unguilata* afforded further batzelladine-like alkaloids that were tested for *in vitro* antimalarial activity;^73^ the pentacyclic spirocycles ptilomycalins E (71), F (72), the mixture of ptilomycalin G and H (73 and 74), crambescidin 800 (75) and fromiamycalin (76) all showed nanomolar potency against *P. falciparum* (IC_50_ 0.23–0.52 μM), but also elevated mammalian cytotoxicity, with CC_50_ values of 0.8–1.6 μM. Unguilicin A (77), an acyclic guanidine alkaloid, exhibited an IC_50_ value of 12.9 μM, but again showed high toxicity to mammalian KB cells. Based on these results, a number of simplified synthetic analogues were prepared as tetrafluoroborate salts, and tested against *T. cruzi* and *L. infantum*.^74^ Representative examples include the tricyclic batzelladine guanidinium ion mimics 78 and 79 and the spiroaminal guanidinium 80, a mimic of ptilomycalin A. IC_50_ values below 10 μM were shown against *T. cruzi* trypomastigotes and *L. infantum* amastigotes in a number of cases; importantly, some compounds showed no mammalian cytotoxicity to the highest tested concentration of 150 μM. As with batzelladine L, 79 caused an increase in the permeability of the *Leishmania* plasma membrane and the levels of ROS, along with depolarization of the mitochondrial membrane. These results suggest there are further opportunities for the development of synthetic batzelladines/ptilomycalins.

A number of sulfur-containing alkaloids have been found to display highly promising anti-protozoal bioactivities. For example, screening of lissoclinotoxin E (81) (along with various other compounds from marine sources) for activity against *T. cruzi*, *L. donovani* and *T. b. brucei* revealed activity against all three parasites with IC_50_ values of 0.7–4.4 μM.^57^ Thiaplakortones A–D (82–85), isolated from the sponge *Plakortis lita*, exhibited sub-micromolar activity against chloroquine-sensitive and chloroquine-resistant lines of *P. falciparum*, with IC_50_ values of 51–650 nM, accompanied by reduced cytotoxicity against the HEK293 cell line,^75^ resulting in exceptional selectivity indices from >62 to >285; saturation of the sulfone-containing ring (as in thiaplakortone A) resulted in a tenfold increase in bioactivity. Being relatively simple from a structural perspective, this family thus represents an attractive synthetic target, and indeed thiaplakortone A and a synthetic analogue 86 were found to present micromolar activity against *T. b. brucei* and *T. cruzi*.^57^

8-Oxo-tryptamine (87), and a mixture of (*E*)- and (*Z*)-bromo-2′-demethyl-3′-*N*-methylaplysinopsin (88), were isolated from the dichloromethane/methanol extract of the sponge *Fascaplysinopsis reticulate*;^76^ both exhibited activity against *P. falciparum* with IC_50_ values around 8 μg mL^−1^. Hyrtiodoline A (89), a further indole alkaloid isolated from a marine sponge of the *Hyrtios* genus, displayed *in vitro* antitrypanosomal properties against *T. b. brucei* (IC_50_ of 15.3 μM), with no cytotoxicity against J774.1 macrophages (CC_50_ >200 μM).^77^

Several polyazacyclic marine natural products have been shown to exhibit promising anti-protozoal properties. For example, caulamidines A (90, Fig. 4) and B (91), obtained from the bryozoan species *Caulibugula intermis*, showed activity against chloroquine-sensitive and chloroquine-resistant strains of *P. falciparum* (IC_50_ 8.3–12.9 μM) with low mammalian cytotoxicity to NCI-60 cells at 40 μM.^78^ The two enantiomers of eudistidine C (92) were isolated through bioactivity-guided fractionation of an ascidian *Eudistoma* sp. extract, and displayed micromolar antimalarial activity against *P. falciparum* (IC_50_ 2.8 and 4.2 μM).^79^ The related alkaloid eudistidine A (93) showed similar antiplasmodial potential (IC_50_ 1.4 μM),^80^ while synthesis of eudistidine C analogues such as 94 resulted in activity against chloroquine-sensitive and chloroquine-resistant strains of *P. falciparum* with IC_50_ values as low as 1.1 μM.^79^ The brevity of this synthetic route (*vide infra*) certainly opens up further opportunities for exploration of this family.

Various exocyclic guanidine and aminopyrimidine alkaloids have been studied with respect to their antiprotozoal properties. Extraction of the sponge *Biemna laboutei* led to the isolation of several such compounds, including netamine K (95) and mirabilin A (96). Both showed promising antimalarial activity with IC_50_ values of 2.4 and 21.0 μM respectively.^81^ Three related guanidines (mirabilin B 97, and hydroxyptilocaulins 98 and 99) were obtained from the bioactive EtOH extract of the marine sponge *Monanchora unguifera*; 97 was found to be active against *L. donovani* promastigotes with (IC_50_ 17.0 μg mL^−1^), while a mixture of 98 and 99 displayed activity against *P. falciparum* (IC_50_ 3.8 μg mL^−1^).^82^ Both 97 and the mixture of 98 and 99 were inactive against fourteen cancer cell lines, suggesting low mammalian cytotoxicity. Further studies on *B. laboutei* afforded other bioactive tricyclic guanidines, including netamines O (100), P (101) and Q (102) which displayed *in vitro* antimalarial activity (IC_50_ of 8.3–33.0 μM).^83^ The bioactivities of this family show high sensitivity to the nature of the various sidechains, and extent of oxidation of the pyrimidine core.

Nortopsentin A (103) is a structurally distinct bis-(indolyl)imidazole alkaloid found in the sponge *Sponhosorites* sp., which displayed antimalarial activity against chloroquine- resistant *P. falciparum* with an IC_50_ value of 0.4 μM, resulting in a selectivity index of 14;^84^ other marine-derived alkaloids studied in this work showed tenfold reduced activity. A number of synthetic approaches to the structurally accessible nortopsentin family have been described, but their wider antiprotozoal profile has yet to be evaluated.^85^

The ingamine alkaloid family consists of two macrocyclic amines built around a bis-piperidine core. Ingamine A (104), (22*S*)-hydroxyingamine A (105) and dihydroingenamine D (106) were isolated from the marine sponge Petrosid Ng5 Sp5; these compounds displayed highly promising activity against chloroquine-sensitive (IC_50_ 72–220 ng mL^−1^) and chloroquine-resistant (IC_50_ 57–140 ng mL^−1^) *P. falciparum*, and showed no cytotoxicity to mammalian cells up to 10 μg mL^−1^.^86^ The dichloromethane/methanol extract of the marine sponge *Neopetrosia proxima* afforded the related bis-piperidine alkaloid neopetrosiamine A (107), which also displayed promising bioactivity against *P. falciparum* (IC_50_ 2.3 μM).^87^ For both of these families, extensive study of antiprotozoal function remains to be explored.

Fractionation of the butanone extract of the marine actinomycete *Marinactinospora thermotolerans* led to isolation of the alkaloids marinacarbolines A–D (108–111), as well as indolactams 13-*N*-demethyl-methylpendolmycin (112) and methylpendolmycin-14-*O*-α-glucoside (113).^88^*In vitro* antimalarial evaluation of these compounds afforded IC_50_ values of 1.9–39 μM for both drug-sensitive and resistant strains of *P. falciparum*, albeit accompanied by significant cytotoxicity. Meridianin A (114), isolated from *Psammopemma* sp., and synthetic analogues 4-methoxymeridianin A (115) and meridoquin (116), were evaluated with regard to their antimalarial potential, affording IC_50_ values of 12–200 μM; only the natural compound 114 was found to be cytotoxic,^89^ while the diethylamino group in 116 resulted in a significant loss of activity compared to the free NH_2_. Meridianins C (117) and G (118), isolated from the tunicate *Aplidium meridianum*,^90^ exhibited improved *in vitro* antimalarial properties (IC_50_ 9.7–14.4 μM), and showed no cytotoxicity against several mammalian and human cell lines up to 25 μM.^91^

Monamphilectine A (119) is a unique β-lactam isonitrile alkaloid isolated from the marine sponge *Hymeniacidon* sp. This compound displayed potent activity against chloroquine-resistant *P. falciparum* (IC_50_ 40 nM).^92^ Finally, lepadins D–F (120–122) were isolated from a marine sponge of the genus *Didemnum*;^93^ activity was displayed against *T. cruzi* amastigotes (IC_50_ 2.2–37 μg mL^−1^), *T. b. brucei* (IC_50_ 0.2–5.6 μg mL^−1^), and against two strains of *P. falciparum* (IC_50_ 0.2–6.1 and 0.4–10 μg mL^−1^). Showing low mammalian cytotoxicity (CC_50_ values from 16 to >30 μg mL^−1^), the lepadins have accordingly stimulated much synthetic interest (*vide infra*). Esterification of the secondary alcohol on the piperidine ring appears important for bioactivity – lepadin D being tenfold less active than lepadins E and F.

In summary, the marine environment is without doubt a rich source of molecularly diverse alkaloids, many displaying useful levels of antiprotozoal behavior. Of course, these compounds are not pre-optimized for use against protozoal parasitic diseases, and their exploitation therefore relies on the development of robust and flexible chemical syntheses, such that structure activity relationships and analogue synthesis can be achieved in a cost- and time-effective manner.

## 4. Synthetic approaches to antiprotozoal marine natural products

This section discusses chemical synthesis approaches to antiprotozoal natural products. In the context of this review, an exhaustive coverage of the natural product syntheses achieved is not possible; instead, representative examples from important family classes are included, with a focus on routes that are potentially scalable, and/or amenable to analogue synthesis, and/or exhibit particular efficiency in accessing challenging synthetic targets.

### 4.1. Indole alkaloids

**Scheme 1:**
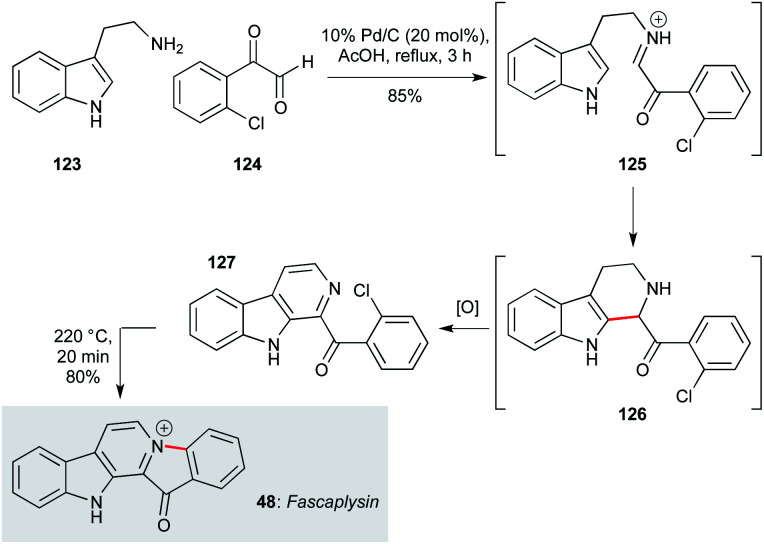
Cyclization/dehydrogenation/N-ring closure cascade sequence to fascaplysin (48) (Bharate and co-workers).^94^

Indole alkaloids comprise one of the largest class of sponge-derived metabolites exhibiting antiprotozoal activity. β-Carboline-type ring systems are common motifs among marine indole alkaloids, as typified by fascaplysin (48, Scheme 1), originally isolated from the marine sponge *Fascapfysinopsis* Bergquist sp.^63,95^ Displaying potent activity against the K1 and NF54 strains of *P. falciparum* (IC_50_ 50 and 34 ng mL^−1^ respectively), several attractive approaches have been described to access this natural product. For example, Bharate and co-workers used a cascade process to form the β-carboline ring system,^94^ building on existing methodology for β-carboline synthesis.^96^ This involved condensation of tryptamine (123) with aromatic glyoxal 124 to give imine 125, which underwent Pictet–Spengler cyclization at C2 of the indole, followed by Pd/C-mediated dehydrogenation/aromatization in acetic acid to yield β-carboline 127. On heating to 220 °C for 20 min, cyclization of 127 produced fascaplysin in 68% overall yield. This concise strategy should be amenable to gram-scale production of fascaplysin and analogues, albeit the thermal cyclization was only performed on 50 mg scale in this work.

**Scheme 2:**
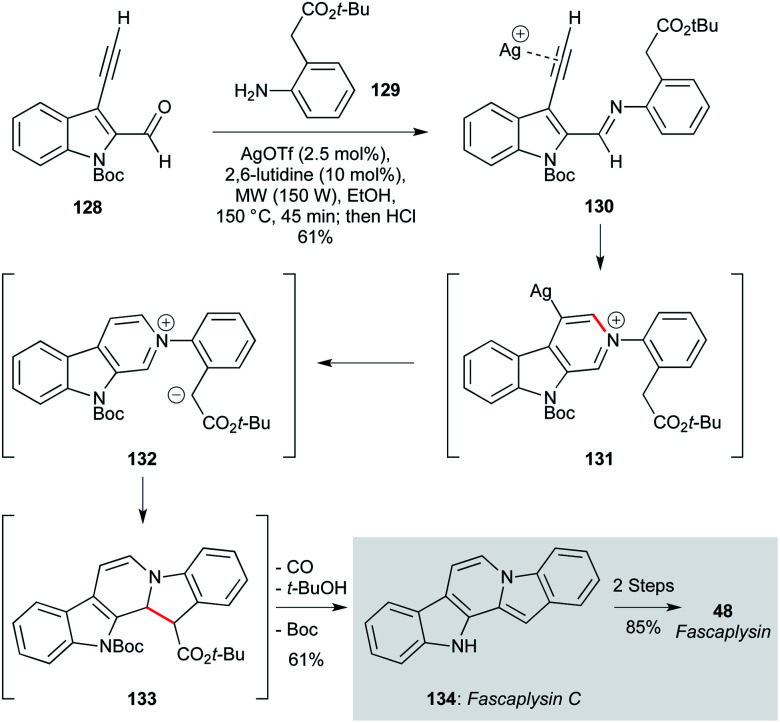
Silver-promoted imine/alkyne cyclization route to fascaplysin (48) (Waldmann and co-workers).^97^

Another appealing route to fascaplysin was reported by Waldmann and co-workers, who used a silver(i)-promoted condensation/cyclization of aldehydes and anilines (Scheme 2).^97^ The silver catalyst promotes cyclization of imine 130 (derived from starting materials 128 and 129) onto the indolyl alkyne, leading to a pyridinium ion which, after protodemetallation, underwent a second cyclisation by the pendent malonate nucleophile to form 132. A final re-aromatization step delivered fascaplysin C (134) in 61% yield, which was converted to fascaplysin (48) in two further steps.

**Scheme 3:**
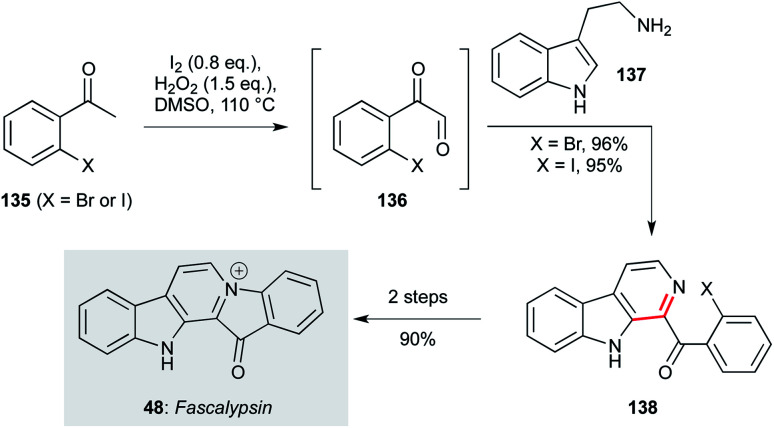
Oxidative condensation/Pictet–Spengler cyclization route to fascaplysin (48) (Zhu and co-workers).^98^

A third approach, disclosed by the Zhu group, again features a cascade coupling protocol for the synthesis of the β-carboline ring system from simple starting materials (Scheme 3).^98^ This route starts with iodination of the haloacetophenone (135), which underwent a Kornblum oxidation with DMSO to give a phenylglyoxal intermediate (136). *In situ* condensation with tryptamine (137), followed by Pictet–Spengler cyclization and oxidation, afforded β-carbolines (138) in an impressive 95/96% yield. These compounds could be converted to fascaplysin in a further two steps.

**Scheme 4:**
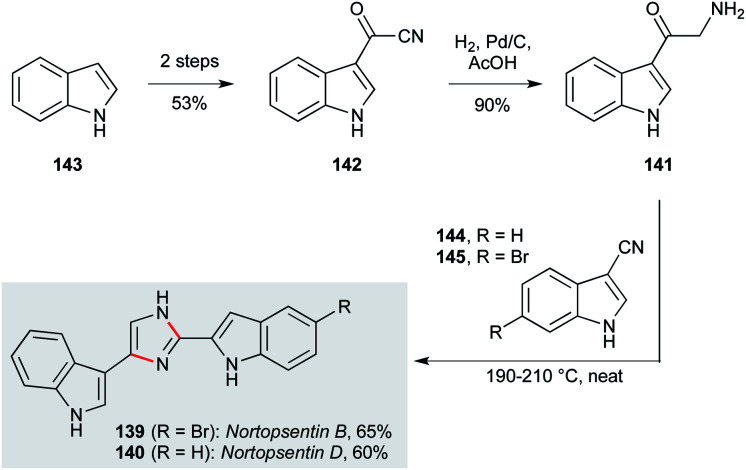
Synthesis of nortopsentins B (139) and D (140) (Miyake and co-workers).^99^

The nortopsentins are a class of imidazole-linked bis-indoles isolated from *Spongosorites ruetzleri*.^100^ Their potent and diverse antiprotozoal activities have made them very attractive compounds as candidates for new drug design, in particular with respect to their antiplasmodial activity (IC_50_ 0.4 μM against *P. falciparum*).^84^ Miyake and co-workers developed an efficient synthesis to access nortopsentins B (139, Scheme 4) and D (140) *via* oxotryptamine 141, which was obtained by a direct hydrogenation of acyl cyanide 142 over Pd/C.^99^ A Markwald imidazole synthesis between 141 and 3-cyanoindole 144 delivered nortopsentin D (140), while reaction of 141 with 6-bromo-3-cyanoindole 145, obtained by bromination of 144*meta* to the indole nitrogen, gave nortopsentin B (139).

**Scheme 5:**
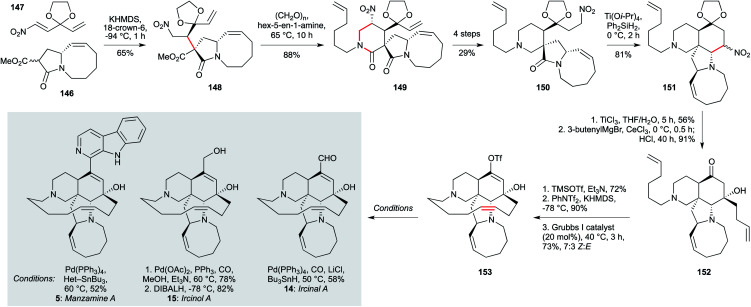
Synthesis of ircinal A (14), iricinol A (15) and manzamine A (5) (Dixon and co-workers).^101^

The manzamine and ircinal alkaloids comprise a structurally complex set of indole natural products isolated from *Haliclona* sp.^102^ and several sponges *Acanthostrongylophora* sp.^50–53,103^ Their challenging architectures might not at first sight seem suited to the preparation of analogues, albeit semi-synthetic modifications have been described.^104^ Nonetheless, a number of synthetic approaches have been reported;^101,105–108^ the recent total synthesis of ircinal A (14, Scheme 5), ircinol A (15) and manzamine A (5) by Dixon *et al.*^101^ is not only the shortest route, but could enable the installation of a variety of heteroaromatics in place of the β-carboline ring system, as well as modification of the macrocyclic alkene region, due to the late-stage introduction of these motifs. These are particularly important targets since, as noted above, manazmine A and ircinol A display potent activity against *L. donovani* (∼1 μg mL^−1^).

From a synthetic perspective, this impressive route features the elegant use of two nitro functionalities to mediate C–C bond formation, and a late-stage ring closing metathesis to assemble the macrocyclic alkene. The synthesis began with the addition of the potassium enolate derived from 5,8-fused ring lactam 146 (itself obtained in five steps from commercially available (*R*)-5-(hydroxymethyl)pyrrolidin-2-one) to nitroalkene 147, to give adduct 148. Having enabled this initial challenging conjugate addition, the nitro group played a second role in a nitro-Mannich reaction with the imine derived from hex-5-en-1-amine and formaldehyde, *in situ* cyclization delivering lactam 149. Having served its purpose, this nitro group was cleaved under reductive conditions, while a second was introduced on the acetal sidechain (150). Under Lewis acid promotion, this enabled a further nitro-Mannich ring closure to tetracycle 151.

In this case, the nitro group was oxidized to the corresponding ketone *via* a McMurray–Nef reaction, stereoselective addition of butenylmagnesium bromide to which afforded tertiary alcohol 152. After temporary alcohol silylation, kinetic enol triflate formation was followed by a moderately *Z*-selective macro-RCM to give enol triflate 153. This compound served asthe direct precursor to ircinal A (14) by palladium-catalyzed formylation, and to ircinol A (15) by palladium-catalyzed carbonylative esterification, followed by reduction. Finally, manzamine A (5) itself was constructed from 153 by Stille coupling with the requisite heteroaryl stannane (18 steps in total, longest linear sequence).

**Scheme 6:**
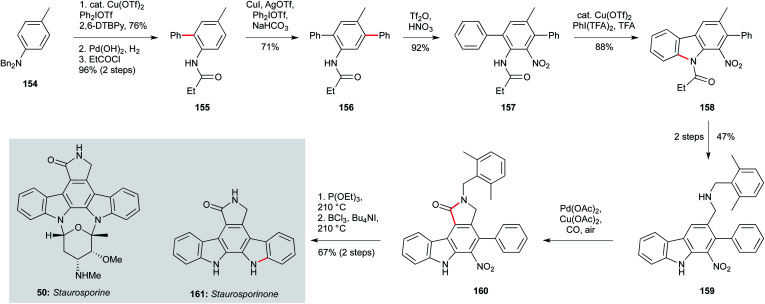
Synthesis of staurosporinone (161) (Gaunt and co-workers).^113^

The indolocarbazole staurosporine family of natural products, isolated from sponge-associated actinomyces^64^ and *Nocardiopsis* sp. K-252,^79,109^ also present a significant synthetic challenge – especially in terms of analogue preparation.^110–112^ While staurosporine (50, Scheme 6) has been shown to exhibit potent activity against *T. b. brucei* (IC_50_ 22 nM), its aglycon staurosporinone (161) has not been tested, and analogues remain elusive. The Gaunt group devised a highly appealing and concise approach that centres on four arene C–H activations – a scalable and modular strategy that could enable the preparation of staurosporinone analogues.^113^ This synthesis begins with the readily available dibenzyl *p*-toluidine (154), which underwent a copper-catalyzed C–H arylation with diphenyliododium triflate to give 155 after a protecting group switch. The amide group was now able to direct a second, *meta*-selective C–H arylation, affording triphenyl 157. The nitrogen substituent required for the second staurosporine indole was then introduced by nitration at the remaining *ortho* position relative to the amide, setting the stage for heterocycle formation. This began with copper-catalyzed C–H amidation to afford 158, and after two steps involving oxidation and reductive amination on the aryl methyl group, amine 159 was subjected to a palladium-catalyzed carbonylative C–H lactamization, delivering the fully- substituted arene core 160. Nitro reduction using triethylphosphite triggered a final arene amination reaction *via* a formal nitrene insertion into the proximal aryl C–H bond. The synthesis of staurosporinone (161) was completed by benzyl cleavage using BCl_3_/*n*-Bu_4_NI. Many of the intermediates in this route were accessed on multigram scale, and the modular and regioselective manner in which the substituents were introduced suggests this approach could hold promise for the wider exploration of staurosporinone biology.

### 4.2. Guanidine alkaloids

#### 4.2.1. Exocyclic guanidines

**Scheme 7:**
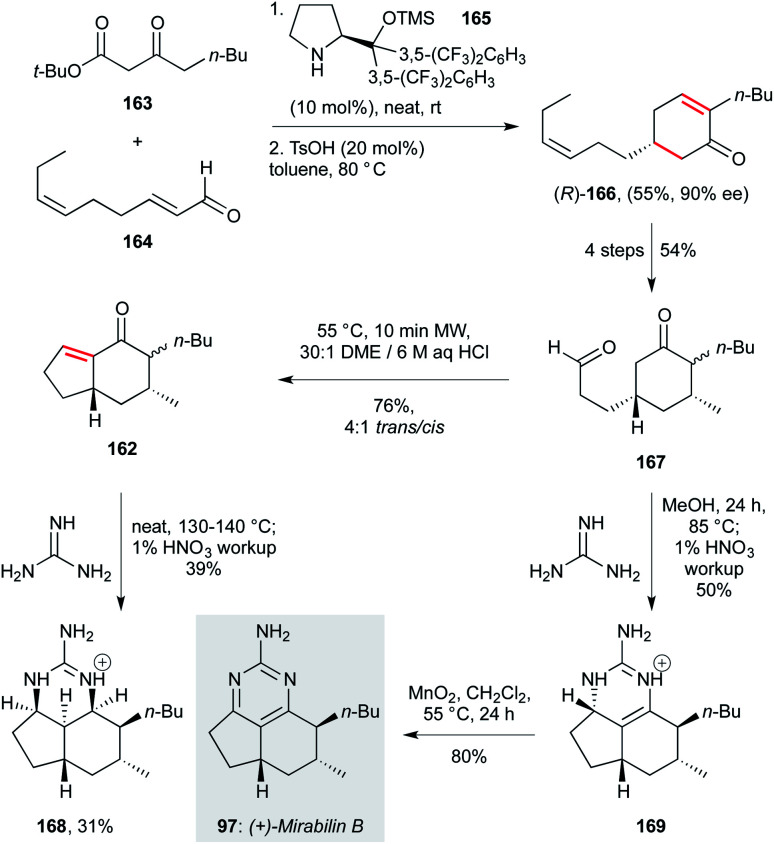
Total synthesis of (+)-mirabilin B (97) (Snider and co-workers).^114^

Marine sponges are a prolific source of polycyclic guanidine-containing compounds, including the tricyclic ptilocaulin, mirabilin and netamine heterocycles.^72,82^ In what constitutes the only total synthesis to date of mirabilin B (31, Scheme 7), Snider and co-workers developed an approach based on the condensation of guanidine with an indenone (162).^114^ This indenone was prepared in six steps, beginning with an asymmetric organocatalytic Michael addition of β-ketoester 163 to enal 164, promoted by the diarylprolinol catalyst 165. Following acid-promoted cyclocondensation, the enantioenriched enone 166 was isolated in respectable yield. A four step sequence involving 1,2-addition of methyllithium and allylic oxygen transposition delivered aldehyde 167 after oxidative cleavage of the alkene sidechain, which underwent anintramolecular aldol/dehydration sequence to give indenone 162, the key intermediate for condensation with guanidine. In the event, this reaction proceeded neat at elevated temperature, with an *in situ* oxidation affording mirabilin B (31) in moderate yield (39%) along with cyclic guanidinium ion 168. More appealingly, treatment of 162 with guanidine in MeOH, followed by oxidation of intermediate 7-*epi*-neoptilocaulin 169 with MnO_2_, gave mirabilin B exclusively, in high yield (80%).

**Scheme 8:**
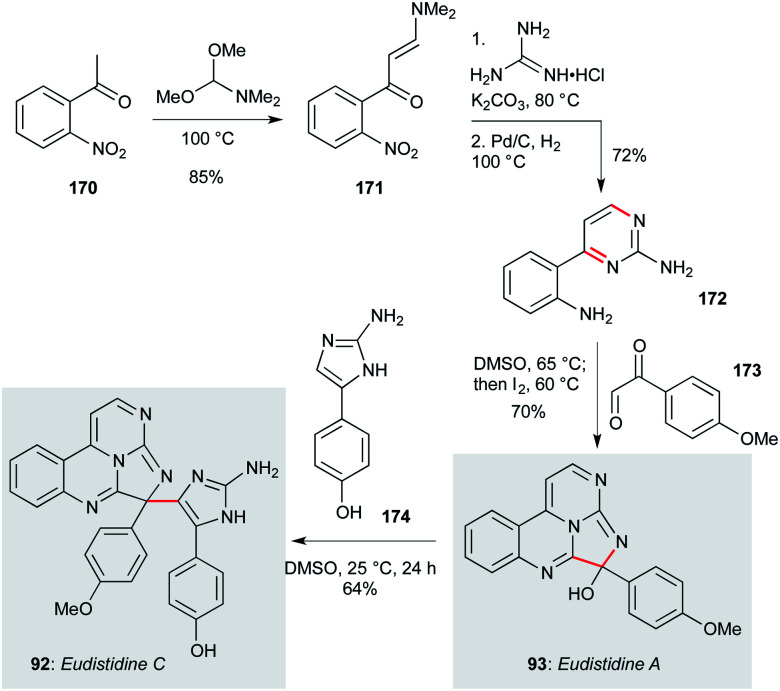
Total synthesis of eudisitidines A (93) and C (92) (Schnermann, Gustafson and co-workers).^79,80^

Eudistidines A (93, Scheme 8) and C (92) feature a polyazaphenanthrene core with aminoimidazole or anisole sidechains, respectively. Isolated from the marine ascidian *Eudistoma* sp. by Schnermann, Gustafson and co-workers,^79,80^ these natural products display low micromolar activity against *P. falciparum*, and have been accessed by a concise synthetic route that enabled the testing of a number of natural product analogues.^79^ This route commenced with 2-nitroacetophenone 170, which underwent condensation with DMF dimethyl acetal to give enaminone 171.^115^ Reaction with guanidine hydrochloride followed by nitro group reduction afforded aminopyrimidine 172. This compound was converted to eudistidine A (93) by condensation/cyclization with aryl glyoxal 173, then *in situ* iodine-mediated oxidation – overall a four step sequence from commercial starting materials. The hemiaminal in eudistidine A represents a convenient site for introduction of other sidechains, including the eudistidine C aminoimidazole ring, which was incorporated by simple stirring of eudistidine A with amine 174 at room temperature. This high-yielding sequence appears well-suited to a broader survey of eudistidine biology.

#### 4.2.2. Endocyclic guanidines

**Scheme 9:**
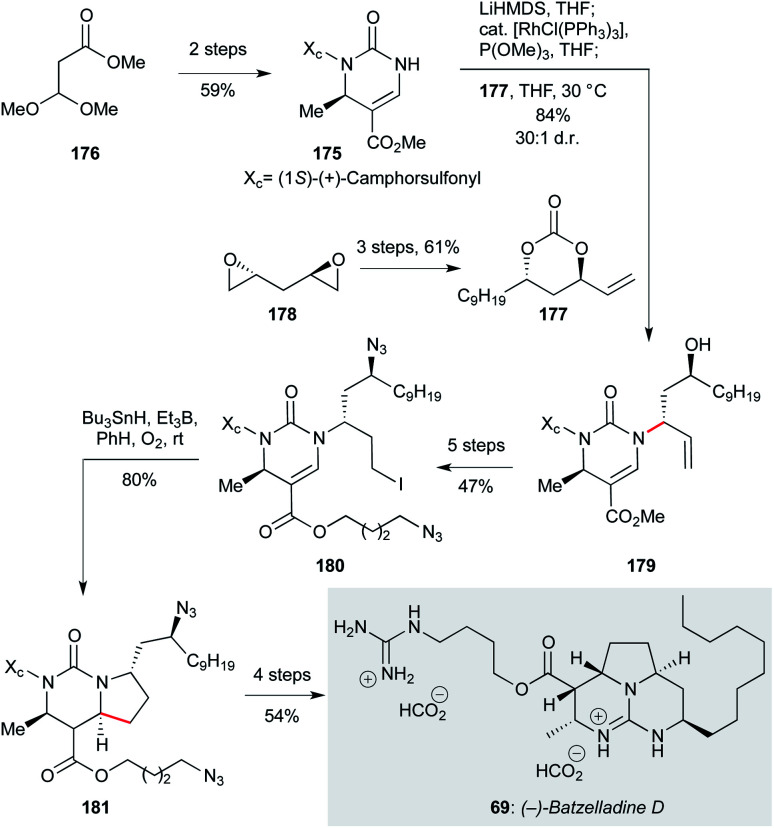
Stereospecific rhodium catalyzed allylic amination/diastereoselective free-radical cyclization in the total synthesis of (−)-batzelladine D (69) (Evans and co-workers).^121^

The antiprotozoal alkaloid (−)-batzelladine D (69, Scheme 9), isolated from the Caribbean sponge *Batzella* sp., is a member of another family of bioactive polycyclic guanidine-containing marine alkaloids.^72^ The tricyclic guanidine core is highly conserved throughout this family, while modifications of the ester side chain elicit different biological activity, which can extend to micromolar or even sub-micromolar levels against *P. falciparum* and *T. cruzi*. Elegant routes have been developed to access a number of members of the batzelladine family;^116–120^ Evans and co-workers recently reported the most concise, enantioselective total synthesis to date of (−)-batzelladine D (69) *via* a 14 step sequence, in an impressive 10% overall yield.^121^ The synthetic strategy is based on a combination of a stereospecific rhodium catalyzed allylic amination, and a diastereoselective free-radical cyclization. The route begins with the preparation of enantiomerically enriched 3,4-dihydropyrimidin-2(1*H*)-one 175 in two steps from commercially available methyl 3,3-dimethoxypropionate 176, *via* acid-catalyzed Biginelli condensation followed by a regioselective sulfonylation with (1*S*)-(+)-camphorsulfonyl chloride (diastereomers separated by column chromatography). Then, rhodium-catalyzed allylic amination of 175 with cyclic carbonate 177, which was prepared in three steps from the *C*2-symmetrical bis-epoxide 178, furnished 179 in 84% yield with high diastereoselectivity (dr >30 : 1). A five step sequence of functional group manipulations afforded alkyl iodide 180, which underwent radical cyclization with tributyltin hydride under triethylborane initiation to give the pyrrolo-[1,2-*f*]-pyrimidine 181 in 80% yield, and 19 : 1 dr (−)-batzelladine D itself (69) was obtained in a further four steps (54% yield).

**Scheme 10:**
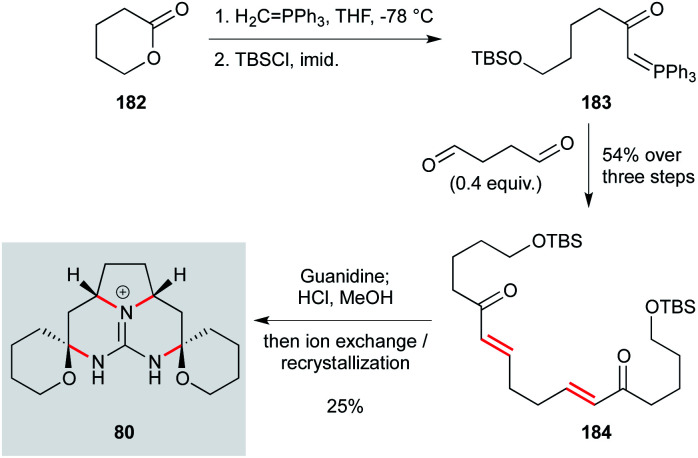
Two-directional synthesis of ptilomycalin analogue 80 (Murphy and co-workers).^122^

While the batzelladine alkaloid family themselves represent challenging synthetic targets, they have also inspired the exploration of simplified natural product analogues that maintain bioactivity. One example is the pentacyclic ptilomycalin A analogue 80 (Scheme 10), which displayed useful levels of activity against *L. infantum* (IC_50_ 8 μM) and low toxicity (SI >19).^74^ Due to its inherent symmetry, this compound can be prepared in just four steps from ∂-valerolactone 182 by ring-opening with methylene triphenylphosphorane, and silylation to afford phosphorane 183.^122^ A double Wittig reaction with succinaldehyde gave bis-enone 184, treatment of which with guanidine effected a tandem Michael addition; acidic workup cleaved the silyl ethers, triggering spiroaminal formation onto the intermediate diketone. This concise synthesis clearly demonstrates the potential of natural products to inspire the development of new and synthetically tractable antiprotozoal agents.

### 4.3. Bromopyrrole alkaloids

**Scheme 11:**
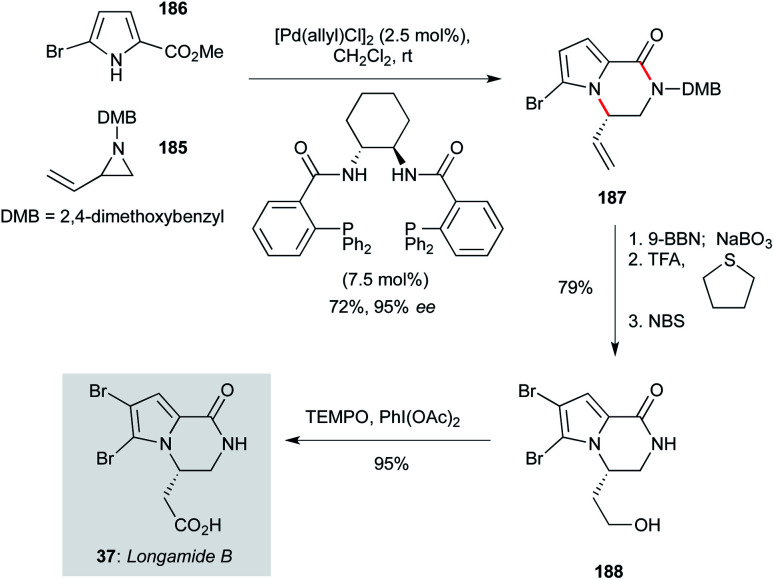
Total synthesis of longamide B (37) (Trost and Dong).^125^

A variety of bromopyrrole-containing sponge metabolites have been tested for their antiprotozoal properties. Among the simplest, and therefore attractive for further development, is longamide B (37, Scheme 11, originally isolated from *Agelas dispar*),^23^ which showed particularly notable activity against *T. b. rhodesiense* and *L. donovani*.^60^ Trost and Dong reported a concise route to access longamide B that centred on an asymmetric palladium-catalyzed addition of A pyrrole nucleophile to a racemic vinyl aziridine (a dynamic kinetic asymmetric transformation, DYKAT).^125^ In the context of the natural product synthesis, reaction of racemic vinyl aziridine 185 (prepared in two steps from 1-amino-3-buten-2-ol) with commercial bromopyrrole 186 in the presence of a chiral palladium catalyst gave the product pyrrolopyrazinone 187 in72% yield and 95% ee. A three step sequence involving alkene hydroboration/oxidation, dimethoxybenzyl cleavage, and pyrrole bromination afforded 188, which could be converted to longamide B (37) by oxidation with TEMPO/bis- acetoxyiodobenzene (BAIB) in high yield. A further publication from the Trost group^126^ demonstrated that a wide range of pyrrole- or indolopyrazinone derivatives should be accessible using this approach, including other natural products.

**Scheme 12:**
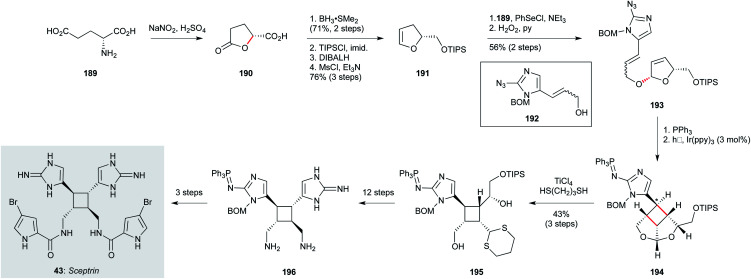
Synthesis of sceptrin (43) (Chen and co-workers).^123,124^

The biosynthesis of sceptrin (43, Scheme 12), a cyclobutane bis-imidazole natural product isolated from *Agelas sceptrum*^127^ likely involves [2+2] dimerization of two simpler aminoimidazole metabolites, triggered by single electron transfer oxidation.^123,124^ Notably, while sceptrin itself has been shown to exhibit notable antiprotozoal activity against *T. b. rhodesiense* and *P. falciparum*, equivalent profiling of its relatives ageliferin and massadine has yet to be carried out. From a synthetic perspective, access to all three of these natural products has been established by the Chen group;^123^ for the purposes of this review, only the route to sceptrin will be described.^124^ This route commences with (d)-glutamic acid (189), which was converted to lactone 190 by anchimeric lactonization. Reduction of the acid, followed by lactone reduction and elimination of the resulting hemiacetal, gave enol ether 191. This electron-rich alkene enabled installation of the alkenylimidazole 192 (prepared in four steps from benzyloxymethyl imidazole) *via* selenoetherification; oxidative elimination of the intermediate selenide afforded dihydrofuran 193. Staudinger reduction of the azide in 193 to the iminophosphine was followed by a tethered photoredox formal [2+2] cycloaddition, which efficiently constructed the sceptrin cyclobutane (194), albeit as a mixture of diastereomers (1.8 : 1) at the indicated ring carbon. Next, transacetalization afforded the cyclobutane 195. At this point, the synthesis suffers from a lengthy endgame sequence involving careful functional group manipulations; most interesting is that the iminophosphine is maintained until the penultimate step of the synthesis. While this route has not yet been applied to analogue synthesis, the efficiency of the tethered photochemical [2+2] suggests simple sceptrin analogues could likely be prepared using this approach.

### 4.4. Thiazine alkaloids

**Scheme 13:**
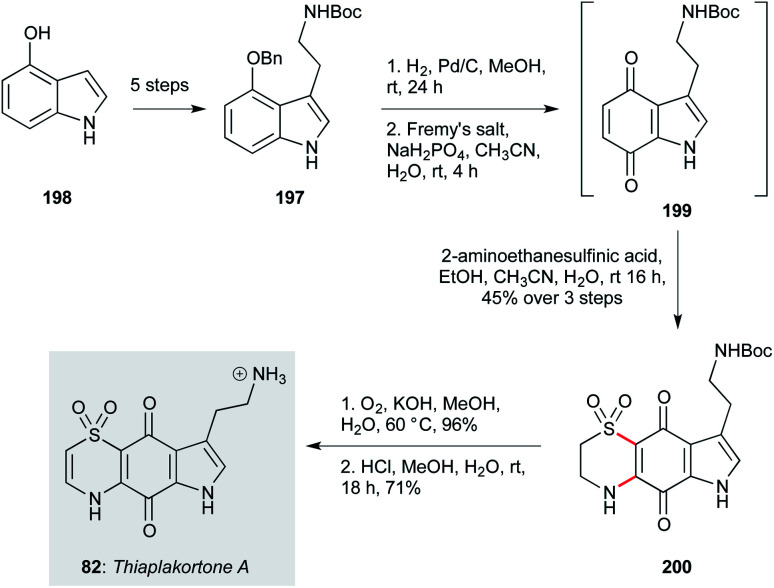
Synthesis of thiaplakortone A (82) (Pouwer and co-workers).^128^

The benzothiazine alkaloids thiaplakortones A–D (82–85) were isolated from the Australian sponge *Plakortis lita* as a result of a high-throughput screen for antimalarial compounds.^75^ The family exhibit sub-micromolar activity against *P. falciparum*, but are also effective against *T. cruzi* and *T. b. brucei*. Pouwer and co-workers developed a simple and efficient synthesis of thiaplakortone A (82, Scheme 13),^128^ and several thiaplakortone analogues.^129^ This began with tryptamine derivative 197, which was readily obtained in five steps from 4-hydroxyindole (198, including only two chromatographic separations). In initial work, this scalable precursor was derivatized by selective methylation on the pyrrole nitrogen, and/or *N*-methylation of the Boc-protected amine sidechain. Benzyl deprotection followed by oxidation with Fremy's salt gave an unstable quinone intermediate 199, which was directly subjected to a double conjugate addition/oxidation sequence upon treatment with 2-aminoethanesulfinic acid, to yield dihydrothiazone dioxide 200 as a separable mixture of regioisomers. Finally, oxidation of the dihydrothiazone and deprotection of the carbamate group afforded thiaplakortone A (82). This efficient and scalable synthesis enabled a further SAR study *via N*-acylations and pyrrole alkylation, which improved the pharmacodynamic properties of the molecule, albeit oral bioavailability remains an identified challenge.^98,129^

### 4.5. Miscellaneous alkaloids

**Scheme 14:**
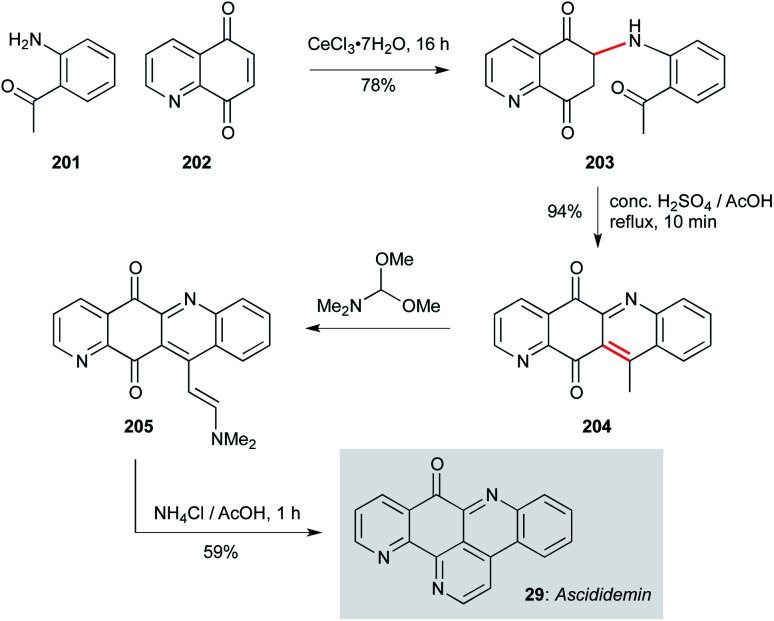
Synthesis of ascididemin (29) (Bracher, Delfourne and co-workers).^131,139^

Ascididemin (29, Scheme 14), a pentacyclic tripyridyl framework isolated from *Polysyncraton echinatum*, displays nanomolar activity against *T. b. brucei*.^130^ With a relatively simple framework, this natural product has been prepared on a number of occasions.^131–138^ However, from a developmental perspective, syntheses that are truly scalable or amenable to analogue synthesis are more challenging. Two approaches are particularly notable in this context; the first is the seminal route from Bracher^131^ which has been developed for analogue synthesis by Delfourne and co-workers due to its convenient and convergent nature.^139^ This chemistry begins with a conjugate addition of 2-amino-acetophenone 201 to 5,8-quinolone 202, presumably promoted by Ce(iii) chelation by the quinolone nitrogen and proximal carbonyl. Treatment of this adduct (203) under acidic conditions effected cyclization/aerobic oxidation to 204. Condensation with DMFDMA gave enamine 205, which was finally converted to ascididemin (29) on reaction with ammonium chloride. In addition to some direct modifications of the natural product, this route was applied to the synthesis of six ascididemin analogues for anticancer profiling, and would clearly translate to an antiprotozoal context.^139^

**Scheme 15:**
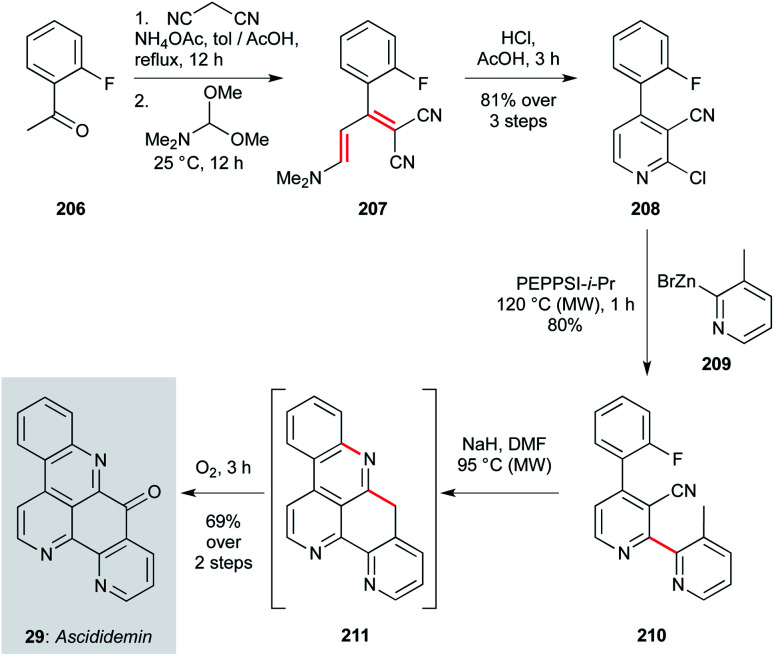
Synthesis of ascididemin (29) (Peterson and co-workers).^137^

The second approach, disclosed by Petersen and co-workers,^137^ features a complementary cross-coupling strategy to assemble the bipyridyl segment of the natural product (Scheme 15). This route begins with Knoevenagel condensation of malononitrile with 2-fluoroacetophenone (206), then formylation with DMFDMA. Treatment of this product (207) with HCl effects direct chloropyridine formation (208) in an impressive 81% yield over the three steps. This sets the stage for Negishi coupling of pyridylzinc 209, forming bypyridine 210 in high yield. Finally, an anionic cyclization cascade is initiated on treatment with sodium hydride, where deprotonation of the 3-methylpyridine motif triggers cyclization onto the proximal nitrile, and then S_N_Ar cyclization onto the fluoroarene. The resultant pentacycle 211 was directly oxidized (O_2_) to ascididemin (29). Comprising just six steps, the modular nature of this synthesis should be readily adapted for analogue exploration.

**Scheme 16:**
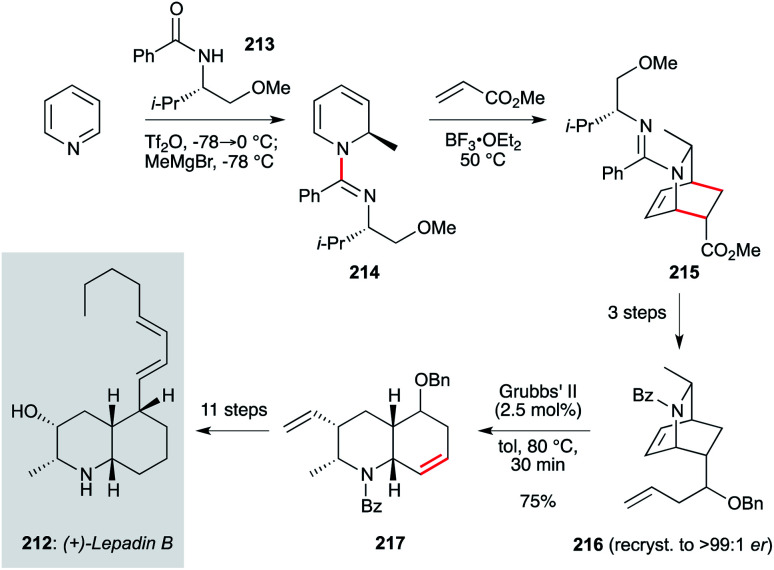
Synthesis of lepadin B (212) (Charette and co-workers).^140^

The lepadin family of decahydroquinoline alkaloids are important targets due to low or sub-microgram mL^−1^ activity against *Trypanosoma* and *Plasmodium* parasites. However, the enantioselective synthesis of these structures, with appropriate sidechain installation, has proven challenging. An attractive and potentially scalable approach that is amenable to analogue synthesis was reported by the Charette group, in their synthesis of lepadin B (212, Scheme 16).^140^ This work begins with pyridine, dearomatization of which by *N*-alkylation with Tf_2_O-activated *N*-benzoyl-*O*-methyl-l-valinol 213, followed by addition of MeMgBr, afforded dihydropyridine 214. This underwent a diastereoselective Diels–Alder reaction to azabicyclo[2.2.2]octane 215, which was advanced to enantiopure amidoalkene 216. This set up an elegant ring-opening/ring-closing metathesis reaction which proceeded on gram scale in a matter of minutes. While a number of steps were needed to advance product 217 to lepadin B, the basis of this route offers a viable entry to this natural product family.

**Scheme 17:**
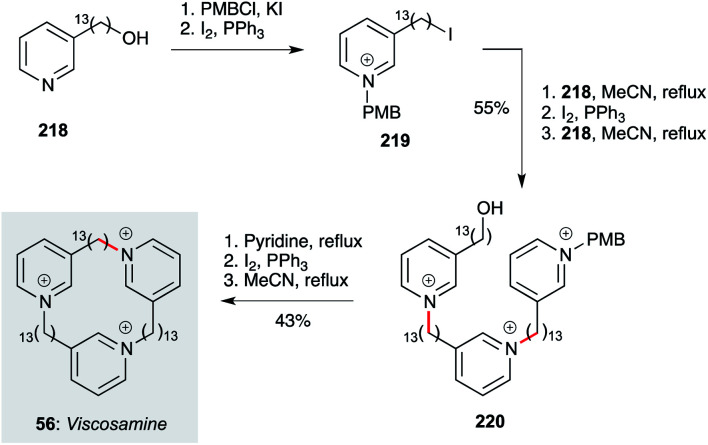
Synthesis of viscosamine (56) (Rodenko, de Koning and co-workers).^70^

Finally, the cyclic tridecylpyridinium alkaloid viscosamine 56 (and non-natural analogues) have been prepared by Rodenko, de Koning and co-workers using a straightforward iterative strategy (Scheme 17).^70^ This begins with pyridine 218, which is converted to iodide 219 after pyridine protection as a *p*-methoxybenzylpyridinium ion. Reaction of 219 with 218, then iodination of the resident alcohol and a further alkylation with 218, delivers trimer 220. The PMB group is cleaved by reaction with excess pyridine, then iodination and cyclization under high dilution (0.0017 M) affords viscosamine 56. Exhibiting sub-micromolar levels of activity against *Leishmania*, *Trypanosoma* and *Plasmodium* parasites, these polar compounds present interesting opportunities for further development.

### 4.6. Opportunities for chemistry

While natural product synthesis can frequently be stimulated by the complexity and challenge of a target molecule, the syntheses described above offer genuine prospects for the study and development of natural product inspired antiprotozoal ‘hit’ compounds. For example, the Rodenko and de Koning synthesis of viscosamine and its synthetic analogues,^70^ which display potent and selective activity against all three parasite genera discussed in this review, can be accessed on multi-100 mg scale; activity against *L. donovani* or *L. infantum* parasites has yet to be evaluated. The same is true of the thiaplakortone family,^128,129^ where synthesis on multi-100 mg scale has been achieved – these compounds and analogues displaying promising activity and selectivity against *P. falciparum* and *T. cruzi*. The relatively simple structures of fascaplysin and nortopsentin, which exhibit low micromolar antimalarial activity and should both be accessible on gram scale using the chemistry described, offer similar potential for development.

More challenging natural products are gradually becoming potential candidates for study, but only where genuinely efficient and modular syntheses have been reported. Probably the best example of this is the Dixon route to the manzamine/ircinol scaffold,^101^ a modular approach which comprises 18 steps in the longest linear sequence, many of which have been carried out on multigram scales. The natural diversity of the manzamine family, matched by the range of biological properties, suggests that this class could be ripe for exploration. For other natural products, challenge still remains – for example, the lepadins are attractive as anti-trypanosomal compounds, but reported syntheses have not yet demonstrated true scalability, while the complex batzelladine family can now be prepared on multi-10 mg scales, enabling a wider exploration of antiprotozoal properties,^74^ but toxicity issues are yet to be overcome. With respect to antileishmanial studies, many alkaloid families have not even been evaluated, which further complicates target selection from a chemical perspective.

## 5. Conclusions

The marine environment offers a wide chemodiversity of natural products, and hence great potential for drug discovery. Many of these compounds likely evolved as chemical defences for the producing organism, but whatever the basis of the anti-protozoal activities and selectivities of marine alkaloids, it is clear that many of these compounds could indeed serve as an inspiration for the development of novel antiprotozoal chemical entities. For this to become reality, the development of efficient total syntheses must underpin the production of new derivatives; recent developments as highlighted in this review show that this goal is within reach. In spite of this potential, few marine alkaloids have yet been tested in animal models of infection, where optimization to improve solubility, stability, pharmacokinetic properties, potency and selectivity become key issues. The elimination of poor-quality compounds, known as pan-assay interference compounds (PAINs), is a further essential consideration to improve the success of drug discovery programmes into malaria and protozoan NTDs. Of the many natural products discussed in this review, a number exhibit activity against more than one parasite type, but the majority remain to be screened against different parasite targets, offering further scope for the discovery of useful bioactive agents. Despite these challenges, the allure of these Nature-optimized leads remains undiminished, and with improved access to both the natural alkaloids and non-natural derivatives, the likelihood of success in the development of natural product-inspired therapies is ever increasing.

## 6. Conflicts of interest

There are no conflicts to declare.

## Supplementary Material

NP-038-D0NP00078G-s001
