# Supplementary material for: Marine alkaloids as bioactive agents against protozoal neglected tropical diseases and malaria
Source: Nat Prod Rep. 2021 Apr 12;38(12):2214–35. doi: 10.1039/d0np00078g (PMC8672869; doi:10.1039/d0np00078g)
Supplement: NP-038-D0NP00078G-s001 [file NP-038-D0NP00078G-s001.pdf]

Electronic Supplementary Material (ESI) for Natural Product Reports.
This journal is © The Royal Society of Chemistry 2021

Marine Alkaloids as Bioactive Agents Against Protozoal Neglected Tropical Diseases and Malaria

Andre G. Tempone,*[a] Pauline Pieper,[b] Samanta E. T. Borborema,[a] Fernanda Thevenard,[c] Joao Henrique G. Lago,[c] Simon L. Croft,*[d] Edward A. Anderson*[b]

[a]*Centre for Parasitology and Mycology, Instituto Adolfo Lutz, 01246-000, São Paulo, Brazil. Email: andre.tempone@ial.sp.gov.br*
[b]*Chemistry Research Laboratory, University of Oxford, 12 Mansfield Road, Oxford OX1 3TA, UK. Email: edward.anderson@chem.ox.ac.uk*
[c]*Centre of Natural Sciences and Humanities, Federal University of ABC, 09210-580, Sao Paulo, Brazil*
[d]*Faculty of Infectious and Tropical Diseases, London School of Hygiene and Tropical Medicine, London WC1E 7HT, UK. Email: simon.croft@lshtm.ac.uk*

Supporting Information

Contents

**Table S1.** *In vitro* bioactivity of marine alkaloids and selected semi-synthetic / synthetic derivatives against *Leishmania* spp., *Trypanosoma* spp., and *Plasmodium falciparum*.	**S2**
**Table S2:** *Plasmodium falciparum* Top hits by IC_{50}: <~1 µg / mL or <~1 µM, and SI>10, ordered by Selectivity Index.	**S7**
**Table S3:** *Trypanosoma* Top hits by IC_{50}: <~1 µg / mL or <~1 µM, and SI>~10, ordered by Selectivity Index.	**S8**
**Table S4:** *Leishmania* Top hits by IC_{50}: <~1 µg / mL or <~1 µM, and SI>~10, ordered by Selectivity Index.	**S8**
**References**	**S9**

Table S1. *In vitro* bioactivity of marine alkaloids and selected semi-synthetic / synthetic derivatives against *Leishmania* spp., *Trypanosoma* spp. and *Plasmodium falciparum*.

[a]*L. a. = Leishmania amazonensis; L. d. = Leishmania donovani; L. i. = Leishmania infantum; L. ma. = Leishmania major; L. me. = Leishmania mexicana; P. b. = Plasmodium berghei; P. f. = Plasmodium falciparum; T. b. b. = Trypanosoma brucei brucei; T. b. r. = Trypanosoma brucei rhodesiense; T. c. = Trypanosoma cruzi.*

NC = no cytotoxicity; ND = not determined; NS = no selectivity (<1). Selectivity Index = CC_{50} value / IC_{50} value; the value must be > 1.

Compd	Natural product	Source	Parasite[a]	IC_{50} value	CC_{50} value / Selectivity Index	Ref.
**1**	renieramycin	*Neopetrosia* sp.	*L. a.*	0.2 µg/mL	2.2 µg/mL / 11.0	43
**2**	araguspongin C	*Haliclona exigua*	*L. d.*	18.6-47.2 µg/mL	34.6 µg/mL / 1.9-NS	44, 45
**3**	8,9-dihydrocoscinamide B	*Conscinoderma* sp.	*L. d.*	>80% inhibition at 10.0 µg/mL	ND	46, 47
**4**	*des-N*-methylxestomanzamine A	Indonesian sponge	*L. d.*	35.0 µg/mL	NC	50
**5**	manzamine A	Indonesian sponge	*L. d.*<br>*P. f.*<br>*P. b.*	0.97 µg/mL<br>0.0045 µg/mL<br>>97% suppression at 100 µmol/kg (*in vivo*)	1.2 µg/mL / 1.3<br>1.2 µg/mL / 266.7<br>ND	50<br><br>51
**6**	8-hydroxymanzamine A	Indonesian sponge	*L. d.*<br>*P. f.*	6.2 µg/mL<br>0.006 µg/mL	1.1 µg/mL / NS<br>1.1 µg/mL / 183	50
**7**	manzamine E	Indonesian sponge	*L. d.*<br>*P. f.*	3.8 µg/mL<br>3.4 µg/mL	NC	50
**8**	manzamine F	Indonesian sponge	*L. d.*<br>*P. f.*	4.2 µg/mL<br>0.78 µg/mL	NC	50
**9**	12,34-oxamanzamine A	Indonesian sponge	*L. d.*<br>*P. f.*	14.0 µg/mL<br>4.76 µg/mL	NC	50
**10**	6-deoxymanzamine X	Indonesian sponge	*L. d.*<br>*P. f.*	3.2 µg/mL<br>1.3 µg/mL	4.76 µg/mL / 1.5<br>4.76 µg/mL / 3.6	50
**11**	manzamine X	*Acanthostronglyophora* sp.	*L. d.*<br>*P. f.*	5.7 µg/mL<br>0.9 µg/mL	NC	50
**12**	manzamine J	*Acanthostronglyophora* sp.	*L. d.*<br>*P. f.*	25.0 µg/mL<br>1.3 µg/mL	NC	52
**13**	manzamine A *N*-oxide	*Acanthostronglyophora* sp.	*L. d.*<br>*P. f.*	1.1 µg/mL<br>0.011 µg/mL	4.2 µg/mL / 3.8<br>4.2 µg/mL / 382	52
**14**	ircinal A	*Acanthostronglyophora* sp.	*L. d.*	4.6 µg/mL	NC	52
**15**	ircinol A	*Acanthostronglyophora* sp.	*L. d.*<br>*P. f.*	0.9 µg/mL<br>2.4 µg/mL	NC	52
**16**	6-hydroxymanzamine E	*Acanthostronglyophora* sp.	*L. d.*<br>*P. f.*	2.5 µg/mL<br>0.78 µg/mL	4.3 µg/mL / 1.7<br>4.3 µg/mL / 5.5	52
**17**	*neo*-kauluamine	*Acanthostronglyophora* sp.	*L. d.*<br>*P. f.*	4.2 µg/mL<br>1.7 µg/mL	NC	52
**18**	manzamine Y	*Acanthostronglyophora* sp.	*L. d.*<br>*P. f.*	1.6 µg/mL<br>0.42 µg/mL	3.9 µg/mL / 2.4<br>3.9 µg/mL / 9.3	53
**19**	12,28-oxamanzamine A	*Acanthostronglyophora* sp.	*L. d.*	7.8 µg/mL	NC	53
**20**	12,28-oxa-8-hydroxymanzamine A	*Acanthostronglyophora* sp.	*L. d.*	24 µg/mL	NC	53

**21**	12,28-oxamanzamine E	*Acanthostronglyophora* sp.	*L. d.*	18.0 µg/mL	NC	53
**22**	8-acetoxymanzamine A	Semi-synthetic	*L. d.*<br>*P. f.*	3.4 µg/mL<br>0.0096 µg/mL	370 µg/mL / 109<br>370 µg/mL / 38542	54
**23**	8,12-diacetoxymanzamine A	Semi-synthetic	*L. d.*<br>*P. f.*	16.0 µg/mL<br>1.3 µg/mL	NC	54
**24**	8-methoxymanzamine A	Semi-synthetic	*L. d.*<br>*P. f.*	5.0 µg/mL<br>0.037 µg/mL	980 µg/mL / 196<br>980 µg/mL / 26486	54
**25**	zamamidine C	*Amphimedon* spp.	*T. b. b.*<br>*P. f.*	0.27 µg/mL<br>0.58 µg/mL	14.1 µg/mL / 52<br>14.1 µg/mL / 24	49
**26**	3,4-dihydro-6-hydroxy-10,11-epoxymanzamine A	*Amphimedon* spp.	*T. b. b.*<br>*P. f.*	0.04 µg/mL<br>0.97 µg/mL	3.6 µg/mL / 90<br>3.6 µg/mL / 3.71	49
**27**	3,4-dihydromanzamine J *N*-oxide	*Amphimedon* spp.	*T. b. b.*<br>*P. f.*	4.4 µg/mL<br>7.0 µg/mL	3.6 µg/mL / NS<br>3.6 µg/mL / NS	49
**28**	12-deoxyascididemin	*P. echinatum*	*T. b. b.*	0.077 µM	7.6 µM / 99	55
**29**	ascididemin	*P. echinatum*	*T. b. b.*	0.032 µM	1.5 µM / 47	55
**30**	eilatin	*P. echinatum*	*T. b. b.*	1.3 µM	ND	55
**31**	convolutamine I	*Amathia tortusa*	*T. b. b.*	1.1 µM	22 µM / 18	56
**32**	convolutamine J	*Amathia tortusa*	*T. b. b.*	13.7 µM	ND	56
**33**	psammaplysin F	*Hyatella* sp.<br>*Pseudoceratina* sp.	*T. c.*<br>*P. f.*	5.6 µM<br>1.9 µM	> 3.5 / NS<br>10.9 µM / 5.7	57<br>58
**34**	psammaplysin G	*Pseudoceratina* sp.	*P. f.*	5.2 µM	18.7 µM / 3.6	57
**35**	psammaplysin H	*Pseudoceratina* sp.	*P. f.*	0.4 µM	> 40 µM / > 97	58
**36**	dibromopalauamine	*Axinella verrucosa*	*T. b. r.*<br>*P. f.*<br>*L. d.*<br>*T. c.*	0.5 µg/mL<br>1.5 µg/mL<br>1.1 µg/mL<br>69.0 µg/mL	4.5 µg/mL / 9<br>4.5µg/mL / 3<br>4.46 µg/mL / 4<br>4.46 µg/mL / NS	60
**37**	longamide B	*Agelas díspar*	*T. b. r.*<br>*P. f.*<br>*L. d.*<br>*T. c.*	1.5 µg/mL<br>7.5 µg/mL<br>3.8 µg/mL<br>33.0 µg/mL	9.9 µg/mL / 6.6<br>9.9 µg/mL / 1.3<br>9.9 µg/mL / 2.6<br>9.9 µg/mL / NS	60
**38**	oroidin	*Axinella* sp. / *Agellas* sp.	*T. b. r.*<br>*P. f.*<br>*L. d.*<br>*T. c.*	12.2 µg/mL<br>7.9 µg/mL<br>15.4 µg/mL<br>> 30.0 µg/mL	76.4 µg/mL / 6.3<br>76.4 µg/mL / 9.7<br>76.4 µg/mL / 4.9<br>ND	60
**39**	hymenidin	*Axinella* sp. / *Agellas* sp.	*T. b. r.*<br>*P. f.*<br>*L. d.*<br>*T. c.*	77.6 µg/mL<br>12.5 µg/mL<br>29.9 µg/mL<br>73.1 µg/mL	75.7 µg/mL / NS<br>75.7 µg/mL / 6.1<br>75.7 µg/mL / 2.5<br>75.7 µg/mL / 1.0	60
**40**	dispacamide B	*Axinella* sp. / *Agellas* sp.	*T. b. r.*<br>*P. f.*<br>*L. d.*<br>*T. c.*	40.1 µg/mL<br>1.3 µg/mL<br>> 90.0 µg/mL<br>> 90.0 µg/mL	> 90 µg/mL / > 2.2<br>> 90 µg/mL / > 69.2<br>ND<br>ND	60
**41**	stevensine	*Axinella* sp. / *Agellas* sp.	*T. b. r.*<br>*P. f.*<br>*L. d.*<br>*T. c.*	25.3 µg/mL<br>4.9 µg/mL<br>75.8 µg/mL<br>>90.0 µg/mL	> 90 µg/mL / > 3.5<br>> 90 µg/mL / > 18.3<br>> 90 µg/mL / > 1.2<br>> 90 µg/mL / > 1.0	60
**42**	spongiacidin B	*Axinella* sp. / *Agellas* sp.	*T. b. r.*<br>*P. f.*<br>*L. d.*<br>*T. c.*	13.5 µg/mL<br>1.1 µg/mL<br>41.6 µg/mL<br>72.2 µg/mL	35.6 µg/mL / 2.6<br>35.6 µg/mL / 32.3<br>35.6 µg/mL / NS<br>35.6 µg/mL / NS	60
**43**	sceptrin	*Axinella* sp. / *Agellas* sp.	*T. b. r.*	9.7 µg/mL	> 90 µg/mL / > 9.3	60

			P. f.<br>*L. d.*<br>*T. c.*	11.1 µg/mL<br>51.6 µg/mL<br>60.0 µg/mL	> 90 µg/mL / > 8.1<br>> 90 µg/mL / > 1.7<br>> 90 µg/mL / > 1.5	
**44**	agelongine	*Axinella* sp. / *Agellas* sp.	*T. b. r.*<br>*P. f.*<br>*L. d.*<br>*T. c.*	49.9 µg/mL<br>11.2 µg/mL<br>43.2 µg/mL<br>>90.0 µg/mL	> 90 µg/mL / > 1.8<br>> 90 µg/mL / > 8.0<br>> 90 µg/mL / > 2<br>> 90 µg/mL / > 1	60
**45**	3-hydroxyacetylindole	*Bacillus pumilus* isolated from *Antipaties* sp.	*T. c.*	20.6 µM	ND	62
**46**	acetyl-β-oxotryptamine	*Bacillus pumilus* isolated from *Antipaties* sp.	*T. c.*	19.4 µM	66 µM / 3.4	62
**47**	3-formylindole	*Bacillus pumilus* isolated from *Antipaties* sp.	*T. c.*	26.9 µM	ND	62
**48**	fascaplysin	*Hyrtios cf. erecta*	*T. b. r.*<br>*P. f.*	0.17 µg/mL<br>0.05 µg/mL	2.5 µg/mL / 14.7<br>2.5 µg/mL / 50	63
**49**	homofascaplysin	*Hyrtios cf. erecta*	*P. f.*	0.014 µg/mL	1.1 µg/mL / 78	63
**50**	staurosporine	*Streptomyces* sp.	*L. ma.*<br>*T. b. b.*	5.3 µM<br>0.02 µM	1.3 µM / 0.25<br>1.3 µM / 65	64
**51**	tryptophol	*Spongia* sp. *and Ircinia* sp.	*L. d.*<br>*T. c.*<br>*T. b. b.*<br>*P. f.*	9.6 µg/mL<br>49.4 µg/mL<br>5.9 µg/mL<br>5.1 µg/mL	63.5 µg/mL / 6.6<br>63.5 µg/mL / 1.3<br>63.5 µg/mL / 11<br>63.5 µg/mL / 13	65
**52**	didemnidine A	*Didemnum* sp.	*T. c.*<br>*T. b. r.*<br>*P. f.*	130.0 µM<br>59.0 µM<br>41.0 µM	24 µM / NS<br>24 µM / NS<br>24 µM / NS	66
**53**	didemnidine B	*Didemnum* sp.	*T. c.*<br>*T. b. r.*<br>*P. f.*	82.0 µM<br>44.0 µM<br>15.0 µM	25 µM / NS<br>25 µM / NS<br>25 µM / 1.7	66
**54**	diazepinomicin	*Micromonospora* sp. from *Aplysina aerophoba*	*T. b. b.*	13.6 µM	ND	67
**55**	paenidigyamycin A	*Paenibacillus* sp.	*L. ma.*<br>*L. d.*<br>*T. b. b.*<br>*P. f.*	0.75 µM<br>7.0 µM<br>0.8 µM<br>9.1 µM	NC	68
**56**	viscosamine	*Haliclona viscosa*	*L. ma.*<br>*L. me.*<br>*T. b. b.*<br>*P. f.*	0.7 µM<br>0.8 µM<br>0.4 µM<br>0.05 µM	26 µM / 37<br>26 µM / 33<br>26 µM / 65<br>26 µM / 520	70
**57**	3-tridecylpyridinium alkaloid	Synthetic	*L. ma*<br>*L. me.*<br>*T. b. b.*<br>*P. f.*	0.8 µM<br>0.2 µM<br>0.2 µM<br>0.07 µM	16 µM / 20<br>16 µM / 80<br>16 µM / 80<br>16 µM / 229	70
**58**	3-tridecylpyridinium alkaloid	Synthetic	*L. ma.*<br>*L. me.*<br>*T. b. b.*<br>*P. f.*	0.6 µM<br>1.1 µM<br>0.5 µM<br>0.2 µM	14 µM / 23<br>14 µM / 13<br>14 µM / 28<br>14 µM / 70	70
**59**	batzelladine A	*Monanchora arbuscula / Clathria calla*	*P. f.*	0.3 µM	2.9 µM / 9.7	71
**60**	norbatzelladine A	*Monanchora arbuscula / Clathria calla*	*P. f.*	0.2 µM	4.7 µM / 24	71
**61**	dinorbatzelladine A	*Monanchora arbuscula / Clathria calla*	*P. f.*	0.9 µM	ND	71
**62**	dinordehydrobatzelladine B	*Monanchora arbuscula / Clathria calla*	*P. f.*	0.8 µM	ND	71

**63**	dihomodehydrobatzelladine C	*Monanchora arbuscula / Clathria calla*	*P. f.*	4.5 µM	ND	71
**64**	clathriadic acid	*Monanchora arbuscula / Clathria calla*	*P. f.*	2.3 µM	ND	71
**65**	ptilomycalin A	*Monanchora arbuscula / Clathria calla*	*P. f.*	0.1 µM	0.1 µM / 1	71
**66**	batzelladine L	*Monanchora arbuscula*	*P. f.*<br>*T. c.*<br>*L. i.*	0.3 µM<br>2.0 µM<br>2.0 µM	< 0.1 µM / NS<br>22 µM / 11<br>22 µM / 11	71<br>72<br>72
**67**	norbatzelladine L	*Monanchora arbuscula*	*P. f.*<br>*T. c.*<br>*L. i.*	0.4 µM<br>7.0 µM<br>2.0 µM	ND<br>85 µM / 12<br>85 µM / 43	71<br>72<br>72
**68**	batzelladine F	*Monanchora arbuscula*	*T. c.*<br>*L. i.*	5.0 µM<br>4.0 µM	10 µM / 2<br>10 µM / 2.5	72
**69**	batzelladine D	*Monanchora arbuscula*	*T. c.*<br>*L. i.*	64.0 µM<br>2.0 µM	130 µM / 2.0<br>130 µM / 65	72
**70**	monalidine A	*Monanchora arbuscula*	*T. c.*<br>*L. i.*	8.0 µM<br>2.0 µM	26 µM / 3.3<br>26 µM / 13	72
**71**	ptilomycalins E	*Monanchora unguiculata*	*P. f.*	0.35 µM	0.85 µM / 2.4	73
**72**	ptilomycalins F	*Monanchora unguiculata*	*P. f.*	0.23 µM	1.61 µM / 7	73
**73** / **74**	ptilomycalin G / H	*Monanchora unguiculata*	*P. f.*	0.46 µM	0.92 µM / 2	73
**75**	crambescidin 800	*Monanchora unguiculata*	*P. f.*	0.52 µM	1.31 µM / 2.5	73
**76**	fromiamycalin	*Monanchora unguiculata*	*P. f.*	0.24 µM	1.17 µM / 5	73
**77**	unguilicin A	*Monanchora unguiculata*	*P. f.*	12.89 µM	7.66 µM / NS	73
**78**	tricyclic guanidinium ion batzelladine mimic	Synthetic	*T. c.*<br>*L. i.*	4.0 µM<br>59.0 µM	84 µM / 21<br>84 µM / 1.4	74
**79**	tricyclic guanidinium ion batzelladine mimic	Synthetic	*T. c.*<br>*L. i.*	9.0 µM<br>2.0 µM (amastigote)<br>34 µM (promastigote)	45 µM / 5<br>45 µM / 22<br>45 µM / 1.3	74
**80**	spiroaminal guanidinium ion	Synthetic	*L. i.*	8.0 µM	> 150 µM / > 19	74
**81**	lissoclinotoxin E	Philippine didemnid ascidian	*T. c.*<br>*L. d.*<br>*L.d.*<br>*T. b. b.*	3.9 µM<br>0.7 (promastigote)<br>4.4 µM (amastigote)<br>0.6 µM	NC	57
**82**	thiaplakortone A	*Plakortis lita*	*T. c.*<br>*T. b. b.*<br>*P. f.*	4.3 µM<br>3.9 µM<br>0.051 µM	NS<br>NS<br>3.9 µM / 76	57<br>57<br>75
**83**	thiaplakortone B	*Plakortis lita*	*P. f.*	0.65 µM	> 40 µM / > 62	75
**84**	thiaplakortone C	*Plakortis lita*	*P. f.*	0.30 µM	> 40 µM / > 133	75
**85**	thiaplakortone D	*Plakortis lita*	*P. f.*	0.28 µM	> 80 µM / > 285	75
**86**	thiaplakortone A analogue	Synthetic	*T. c.*<br>*T. b. b.*	3.5 µM<br>0.7 µM	ND	57
**87**	8-oxo-tryptamine	*Fascaplysinopsis reticulata*	*P. f.*	8.8 µg/mL	ND	76
**(*E*/*Z*)-88**	(*E*)/(*Z*)-bromo-2'-demethyl-3'-*N*-methylaplysinopsin	*Fascaplysinopsis reticulata*	*P. f.*	8.0 µg/mL	ND	76
**89**	hyrtiodoline A	*Hyrtios* sp.	*T. b. b.*	15.2 µM	> 200 µM / > 13	77
**90**	caulidamine A	*Caulibugula intermis*	*P. f.*	8.3 µM	ND	78
**91**	caulidamine B	*Caulibugula intermis*	*P. f.*	12.9 µM	ND	78

**92**	eudistidine C	*Eudistoma* sp.	*P. f.*	2.8-4.2 µM	NC	79
**93**	eudistidine A	*Eudistoma* sp.	*P. f.*	1.4 µM	NC	79, 80
**94**	eudistidine C analogue	synthetic	*P. f.*	1.1 µM	NC	79
**95**	netamine K	*Biemna laboutei*	*P. f.*	2.4 µM	> 1 µM / NS	81
**95**	mirabilin A	*Biemna laboutei*	*P. f.*	21.0 µM	> 1 µM / NS	81
**97**	mirabilin B	*Monanchora unguifera*	*L. d.*	17.6 µg/mL	NC	82
**98**	1,8a;8b,3a-didehydro-8b-hydroxyptilocaulin	*Monanchora unguifera*	*P. f.*	3.8 µg/mL	NC	82
**99**	1,8a;8b,3a-didehydro-8a hydroxyptilocaulin	*Monanchora unguifera*	*P. f.*	3.8 µg/mL	NC	82
**100**	netamine O	*Biemna laboutei*	*P. f.*	17.0 µM	> 0.01 µM / NS	83
**101**	netamine P	*Biemna laboutei*	*P. f.*	33.0 µM	> 0.01 µM / NS	83
**102**	netamine Q	*Biemna laboutei*	*P. f.*	8.4 µM	> 0.01 µM / NS	83
**103**	nortopsentin A	*Spongosorites*	*P. f.*	0.4 µM	6.6 µM / 14	84
**104**	ingamine A	*Petrosid Ng5 Sp5*	*P. f.*<br>*L. d.*	0.09 µg/mL<br>5.98 µg/mL	> 10 µg/mL / > 111<br>> 10 µg/mL / > 1.7	86
**105**	hydroxyingamine A	*Petrosid Ng5 Sp5*	*P. f.*<br>*L. d.*	0.22 µg/mL<br>5.83 µg/mL	> 10 µg/mL / > 45<br>> 10 µg/mL / > 1.7	86
**106**	dihydroingenamine D	*Petrosid Ng5 Sp5*	*P. f.*<br>*L. d.*	0.078 µg/mL<br>3.12 µg/mL	> 10 µg/mL / > 128<br>> 10 µg/mL / > 3.2	86
**107**	neopetrosiamine A	*Neopetrosia proxima*	*P. f.*	2.3 µM	ND	87
**108**	marinacarboline A	*Marinactinospora thermotolerans*	*P. f.*	1.9 µM	> 50 µM / > 26	88
**109**	marinacarboline B	*Marinactinospora thermotolerans*	*P. f.*	15.6 µM	> 50 µM / > 3.2	88
**110**	marinacarboline C	*Marinactinospora thermotolerans*	*P. f.*	3.4 µM	> 50 µM / > 15	88
**111**	marinacarboline D	*Marinactinospora thermotolerans*	*P. f.*	3.6 µM	> 50 µM / > 14	88
**112**	13-N-demethyl-methylpendolmycin	*Marinactinospora thermotolerans*	*P. f.*	18.7 µM	> 50 µM / > 2.7	88
**113**	methylpendolmy-cin-14-O-α-glucoside	*Marinactinospora thermotolerans*	*P. f.*	5.0 µM	> 50 µM / > 5	88
**114**	meridianin A	*Psammopemma* sp. / *Aplidium meridianum*	*P. f.*	12 µM	15.0 µM / 1.2	89
**115**	4-methoxymeridianin A	synthetic	*P. f.*	40 µM	> 420 µM / > 10	89
**116**	meridoquin	synthetic	*P. f.*	200 µM	> 333 µM / > 1.7	89
**117**	meridianin C	*Aplidium meridianum*	*P. f.*	14.4 µM	> 87 µM / > 6.0 µM	91
**118**	meridianin G	*Aplidium meridianum*	*P. f.*	9.7 µM	> 119 µM / > 11 µM	91
**119**	monamphilectine A	*Hymeniacidon* sp.	*P. f.*	0.04 µM	ND	92
**120**	lepadin D	*Didemnum* sp.	*T. b. r.*<br>*T. c.*<br>*P. f.*	5.6 µg/mL<br>37.2 µg/mL<br>10.0 µg/mL	> 30 µg/mL / > 5.4<br>> 30 µg/mL / NS<br>> 30 µg/mL / > 3	93
**121**	lepadin E	*Didemnum* sp.	*T. b. r.*<br>*T. c.*<br>*P. f.*	0.38 µg/mL<br>2.2 µg/mL<br>0.9 µg/mL	16.2 µg/mL / 43<br>16.2 µg/mL / 7.4<br>16.2 µg/mL / 18	93
**122**	lepadin F	*Didemnum* sp.	*T. b. r.*<br>*T. c.*<br>*P. f.*	0.23 µg/mL<br>2.6 µg/mL<br>0.3 µg/mL	18.43 µg/mL / 80<br>18.43 µg/mL / 7.1<br>18.43 µg/mL / 61	93

Table S2: *Plasmodium falciparum* Top hits by IC_{50}: <~1 µg / mL or <~1 µM, and SI>10, ordered by Selectivity Index.

Compound	Natural product	*Source*	IC_{50} value	SI	Ref.
**8**	manzamine F	*Indonesian sponge*	0.78 µg/mL	NC	48
**11**	manzamine X	*Acanthostronglyophora* sp.	0.9 µg/mL	NC	48
**94**	eudistidine C analogue	synthetic	1.1 µM	NC	79
**22**	8-acetoxymanzamine A	Semi-synthetic	0.0096 µg/mL	38542	54
**24**	8-methoxymanzamine A	Semi-synthetic	0.037 µg/mL	26486	54
**56**	viscosamine	*Haliclona viscosa*	0.05 µM	520	70
**13**	manzamine A N-oxide	*Acanthostronglyophora* sp.	0.011 µg/mL	382	52
**85**	thiaplakortone D	*Plakortis lita*	0.28 µM	>285	75
**5**	manzamine A	*Indonesian sponge*	0.0045 µg/mL	266.7	48
**57**	3-tridecylpyridinium alkaloid	Synthetic	0.07 µM	229	70
**6**	8-hydroxymanzamine A	Indonesian sponge	0.006 µg/mL	183	48
**84**	thiaplakortone C	*Plakortis lita*	0.30 µM	>133	75
**106**	dihydroingenamine D	*Petrosid Ng5 Sp5*	0.078 µg/mL	>128	86
**104**	ingamine A	*Petrosid Ng5 Sp5*	0.09 µg/mL	>11	86
**35**	psammaplysin H	*Pseudoceratina* sp.	0.4 µM	>97	59
**49**	homofascaplysin	*Hyrtios cf. erecta*	0.014 µg/mL	78	63
**82**	thiaplakortone A	*Plakortis lita*	0.051 µM	76	75
**58**	3-tridecylpyridinium alkaloid	Synthetic	0.2 µM	70	70
**83**	thiaplakortone B	*Plakortis lita*	0.65 µM	>62	75
**122**	lepadin F	*Didemnum* sp.	0.3 µg/mL	61	93
**48**	fascaplysin	*Hyrtios cf. erecta*	0.05 µg/mL	50	63
**105**	hydroxyingamine A	*Petrosid Ng5 Sp5*	0.22 µg/mL	> 45	86
**25**	zamamidine C	*Amphimedon* spp.	0.58 µg/mL	24	47
**121**	lepadin E	*Didemnum* sp.	0.9 µg/mL	18	93
**103**	nortopsentin A	*Spongosorites*	0.4 µM	14	84
**67**	norbatzelladine L	*Monanchora arbuscula*	0.4 µM	ND	71
**119**	monamphilectine A	*Hymeniacidon* sp.	0.04 µM	ND	92

Table S3: *Trypanosoma* Top hits by IC_{50}: <~1 µg / mL or <~1 µM, and SI>~10, ordered by Selectivity Index.

**Compd**	Natural product	*Source*	*Parasitea*	IC_{50} value	SI	Ref.
**28**	12-deoxyascididemin	*P. echinatum*	*T. b. b.*	0.077 µM	99	54
**26**	3,4-dihydro-6-hydroxy-10,11-epoxymanzamine A	*Amphimedon* spp.	*T. b. b.*	0.04 µg/mL	90	47
**57**	3-tridecylpyridinium alkaloid	Synthetic	*T. b. b.*	0.2 µM	80	70
**122**	lepadin F	*Didemnum* sp.	*T. b. r.*<br>*T. c.*	0.23 µg/mL<br>2.6 µg/mL	80<br>7.1	93
**50**	staurosporine	*Streptomyces* sp.	*T. b. b.*	0.02 µM	65	64
**56**	viscosamine	*Haliclona viscosa*	*T. b. b.*	0.4 µM	65	70
**25**	zamamidine C	*Amphimedon* spp.	*T. b. b.*	0.27 µg/mL	52	47
**29**	ascididemin	*P. echinatum*	*T. b. b.*	0.032 µM	47	55
**121**	lepadin E	*Didemnum* sp.	*T. b. r.*<br>*T. c.*	0.38 µg/mL<br>2.2 µg/mL	43<br>7.4	93
**48**	fascaplysin	*Hyrtios cf. erecta*	*T. b. r.*	0.17 µg/mL	14.7	63
**58**	3-tridecylpyridinium alkaloid	Synthetic	*T. b. b.*	0.5 µM	28	70
**36**	dibromopalauamine	*Axinella verrucosa*	*T. b. r.*<br>*T. c.*	0.5 µg/mL<br>69.0 µg/mL	9<br>NS	60
**86**	thiaplakortone A analogue	Synthetic	*T. c.*<br>*T. b. b.*	3.5 µM<br>0.7 µM	ND<br>ND	57

Table S4: *Leishmania* Top hits by IC_{50}: <~1 µg / mL or <~1 µM, and SI>~10, ordered by Selectivity Index.

**Compd**	Natural product	*Source*	*Parasitea*	IC_{50} value	SI	Ref.
**15**	ircinol A	*Acanthostronglyophora* sp.	*L. d.*	0.9 µg/mL	NC	52
**57**	3-tridecylpyridinium alkaloid	Synthetic	*L. ma*<br>*L. me.*	0.8 µM<br>0.2 µM	20<br>80	70
**56**	viscosamine	*Haliclona viscosa*	*L. ma.*<br>*L. me.*	0.7 µM<br>0.8 µM	37<br>33	70
**58**	3-tridecylpyridinium alkaloid	Synthetic	*L. ma.*<br>*L. me.*	0.6 µM<br>1.1 µM	23<br>13	70
**1**	renieramycin	*Neopetrosia* sp.	*L. a.*	0.2 µg/mL	11.0	41

References *(note: Reference numbering corresponds to that in the review article).*

43 Y. Nakao, T. Shiroiwa, S. Murayama, S. Matsunaga, Y. Goto, Y. Matsumoto and N. Fusetani, *Mar. Drugs*, 2004, **2**, 55-62.

44 Y. Venkateswarlu, M. V. R. Reddy and J. V. Rao, *J. Nat. Prod.*, 1994, **57**, 1283-1285.

45 A. Dube, N. Singh, A. Saxena and V. Lakshmi, *Parasitology Res.*, 2007, **101**, 317-324.

46 H. R. Bokesch, L. K. Pannell, T. C. McKee and M. R. Boyd, *Tetrahedron Lett.*, 2000, **41**, 6305-6308.

47 L. Gupta, A. Talwar, Nishi, S. Palne, S. Gupta and P. M. S. Chauhan, *Bioorg. Med. Chem. Lett.*, 2007, **17**, 4075-4079.

49 M. Yamada, Y. Takahashi, T. Kubota, J. Fromont, A. Ishiyama, K. Otoguro, H. Yamada, S. Ōmura and J. i. Kobayashi, *Tetrahedron*, 2009, **65**, 2313-2317.

50 K. V. Rao, B. D. Santarsiero, A. D. Mesecar, R. F. Schinazi, B. L. Tekwani and M. T. Hamann, *J. Nat. Prod.*, 2003, **66**, 823-828.

51 K. Ang, M. Holmes and U. Kara, *Parasitology Res.*, 2001, **87**, 715-721.

52 K. V. Rao, N. Kasanah, S. Wahyuono, B. L. Tekwani, R. F. Schinazi and M. T. Hamann, *J. Nat. Prod.*, 2004, **67**, 1314-1318.

53 K. V. Rao, M. S. Donia, J. Peng, E. Garcia-Palomero, D. Alonso, A. Martinez, M. Medina, S. G. Franzblau, B. L. Tekwani, S. I. Khan, S. Wahyuono, K. L. Willett and M. T. Hamann, *J. Nat. Prod.*, 2006, **69**, 1034-1040.

54 A. G. Shilabin, N. Kasanah, B. L. Tekwani and M. T. Hamann, *J. Nat. Prod.*, 2008, **71**, 1218-1221.

55 Y. Feng, R. A. Davis, M. L. Sykes, V. M. Avery, A. R. Carroll, D. Camp and R. J. Quinn, *Tetrahedron Lett.*, 2010, **51**, 2477-2479.

56 R. A. Davis, M. Sykes, V. M. Avery, D. Camp and R. J. Quinn, *Bioorg. Med. Chem.*, 2011, **19**, 6615-6619.

57 B. Zulfiqar, A. Jones, M. Sykes, T. Shelper, R. Davis and V. Avery, *Molecules*, 2017, **22**, 1715-1715.

58 X. Yang, R. A. Davis, M. S. Buchanan, S. Duffy, V. M. Avery, D. Camp and R. J. Quinn, *J. Nat. Prod.*, 2010, **73**, 985-987.

59 M. Xu, K. T. Andrews, G. W. Birrell, T. L. Tran, D. Camp, R. A. Davis and R. J. Quinn, *Bioorg. Med. Chem. Lett.*, 2011, **21**, 846-848.

60 F. Scala, E. Fattorusso, M. Menna, O. Taglialatela-Scafati, M. Tierney, M. Kaiser and D. Tasdemir, *Mar. Drugs*, 2010, **8**, 2162-2174.

62 S. Martínez-Luis, J. F. Gómez, C. Spadafora, H. M. Guzmán and M. Gutiérrez, *Molecules*, 2012, **17**, 11146-11155.

63 G. Kirsch, G. M. König, A. D. Wright and R. Kaminsky, *J. Nat. Prod.*, 2000, **63**, 825-829.

64 S. M. Pimentel-Elardo, S. Kozytska, T. S. Bugni, C. M. Ireland, H. Moll and U. Hentschel, *Mar. Drugs*, 2010, **8**, 373-380.

65 I. Orhan, B. Şener, M. Kaiser, R. Brun and D. Tasdemir, *Mar. Drugs*, 2010, **8**, 47-58.

66 R. Finlayson, A. N. Pearce, M. J. Page, M. Kaiser, M.-L. Bourguet-Kondracki, J. L. Harper, V. L. Webb and B. R. Copp, *J. Nat. Prod.*, 2011, **74**, 888-892.

67 U. R. Abdelmohsen, M. Szesny, E. M. Othman, T. Schirmeister, S. Grond, H. Stopper and U. Hentschel, *Mar. Drugs*, 2012, **10**, 2208-2221.

68 E. Osei, S. Kwain, G. Mawuli, A. Anang, K. Owusu, M. Camas, A. Camas, M. Ohashi, C.-N. Alexandru-Crivac, H. Deng, M. Jaspars and K. Kyeremeh, *Mar. Drugs*, 2019, **17**, 1-13.

69 B. Rodenko, M. I. Al-Salabi, I. A. Teka, W. Ho, N. El-Sabbagh, J. A. M. Ali, H. M. S. Ibrahim, M. J. Wanner, G.-J. Koomen and H. P. de Koning, *ACS Med. Chem. Lett.*, 2011, **2**, 901-906.

71 R. m. Laville, O. P. Thomas, F. Berrué, D. Marquez, J. Vacelet and P. Amade, *J. Nat. Prod.*, 2009, **72**, 1589-1594.

72 M. F. C. Santos, P. M. Harper, D. E. Williams, J. T. Mesquita, É. G. Pinto, T. A. da Costa-Silva, E. Hajdu, A. G. Ferreira, R. A. Santos, P. J. Murphy, R. J. Andersen, A. G. Tempone and R. G. S. Berlinck, *J. Nat. Prod.*, 2015, **78**, 1101-1112.

73 P.-E. Campos, J.-L. Wolfender, E. F. Queiroz, L. Marcourt, A. Al-Mourabit, M. Frederich, A. Bordignon, N. De Voogd, B. Illien and A. Gauvin-Bialecki, *J. Nat. Prod.*, 2017, **80**, 1404-1410.

74 L. F. Martins, J. T. Mesquita, E. G. Pinto, T. A. Costa-Silva, S. E. T. Borborema, A. J. Galisteo Junior, B. J. Neves, C. H. Andrade, Z. A. Shuhaib, E. L. Bennett, G. P. Black, P. M. Harper, D. M. Evans, H. S. Fituri, J. P. Leyland, C. Martin, T. D. Roberts, A. J. Thornhill, S. A. Vale, A. Howard-Jones, D. A. Thomas,

H. L. Williams, L. E. Overman, R. G. S. Berlinck, P. J. Murphy and A. G. Tempone, *J. Nat. Prod.*, 2016, **79**, 2202-2210.

75 R. A. Davis, S. Duffy, S. Fletcher, V. M. Avery and R. J. Quinn, *J. Org. Chem.*, 2013, **78**, 9608-9613.

76 P.-E. Campos, E. Pichon, C. Moriou, P. Clerc, R. Trépos, M. Frederich, N. De Voogd, C. Hellio, A. Gauvin-Bialecki and A. Al-Mourabit, *Mar. Drugs*, 2019, **17**, 167-167.

77 N. H. Shady, M. A. Fouad, S. Ahmed, S. M. Pimentel-Elardo, J. R. Nodwell, M. S. Kamel and U. R. Abdelmohsen, *J. Antibiot.*, 2018, **71**, 1036-1039.

78 D. J. Milanowski, N. Oku, L. K. Cartner, H. R. Bokesch, R. T. Williamson, J. Saurí, Y. Liu, K. A. Blinov, Y. Ding, X.-C. Li, D. Ferreira, L. A. Walker, S. Khan, M. T. Davies-Coleman, J. A. Kelley, J. B. McMahon, G. E. Martin and K. R. Gustafson, *Chem. Sci.*, 2018, **9**, 307-314.

79 S. T. S. Chan, R. R. Nani, E. A. Schauer, G. E. Martin, R. T. Williamson, J. Saurí, A. V. Buevich, W. A. Schafer, L. A. Joyce, A. K. L. Goey, W. D. Figg, T. T. Ransom, C. J. Henrich, T. C. McKee, A. Moser, S. A. MacDonald, S. Khan, J. B. McMahon, M. J. Schnermann and K. R. Gustafson, *J. Org. Chem.*, 2016, **81**, 10631-10640.

80 S. T. S. Chan, P. R. Patel, T. R. Ransom, C. J. Henrich, T. C. McKee, A. K. L. Goey, K. M. Cook, W. D. Figg, J. B. McMahon, M. J. Schnermann and K. R. Gustafson, *J. Am. Chem. Soc.*, 2015, **137**, 5569-5575.

81 E. Gros, A. Al-Mourabit, M.-T. Martin, J. Sorres, J. Vacelet, M. Frederich, M. Aknin, Y. Kashman and A. Gauvin-Bialecki, *J. Nat. Prod.*, 2014, **77**, 818-823.

82 H.-m. Hua, J. Peng, F. R. Fronczek, M. Kelly and M. T. Hamann, *Bioorg. Med. Chem.*, 2004, **12**, 6461-6464.

83 E. Gros, M.-T. Martin, J. Sorres, C. Moriou, J. Vacelet, M. Frederich, M. Aknin, Y. Kashman, A. Gauvin-Bialecki and A. Al-Mourabit, *Chem. Biodivers.*, 2015, **12**, 1725-1733.

84 S. Alvarado, B. F. Roberts, A. E. Wright and D. Chakrabarti, *Antimicrob. Agents Chemother.*, 2013, **57**, 2362-2364.

86 M. Ilias, M. Ibrahim, S. Khan, M. Jacob, B. Tekwani, L. Walker and V. Samoylenko, *Planta Med.*, 2012, **78**, 1690-1697.

87 X. Wei, K. Nieves and A. D. Rodríguez, *Bioorg. Med. Chem. Lett.*, 2010, **20**, 5905-5908.

88 H. Huang, Y. Yao, Z. He, T. Yang, J. Ma, X. Tian, Y. Li, C. Huang, X. Chen, W. Li, S. Zhang, C. Zhang and J. Ju, *J. Nat. Prod.*, 2011, **74**, 2122-2127.

89 M. D. Lebar, K. N. Hahn, T. Mutka, P. Maignan, J. B. McClintock, C. D. Amsler, A. van Olphen, D. E. Kyle and B. J. Baker, *Bioorg. Med. Chem.*, 2011, **19**, 5756-5762.

91 R. R. Yadav, S. I. Khan, S. Singh, I. A. Khan, R. A. Vishwakarma and S. B. Bharate, *Eur. J. Med. Chem.*, 2015, **98**, 160-169.

92 E. Avilés and A. D. Rodríguez, *Org. Lett.*, 2010, **12**, 5290-5293.

93 A. D. Wright, E. Goclik, G. M. König and R. Kaminsky, *J. Med. Chem.*, 2002, **45**, 3067-3072.
